# Selection and Phylogenetics of Salmonid MHC Class I: Wild Brown Trout (*Salmo trutta*) Differ from a Non-Native Introduced Strain

**DOI:** 10.1371/journal.pone.0063035

**Published:** 2013-05-07

**Authors:** Brian O'Farrell, John A. H. Benzie, Phil McGinnity, Elvira de Eyto, Eileen Dillane, James Coughlan, Tom F. Cross

**Affiliations:** 1 Environmental Research Institute, University College Cork, Cork, Ireland; 2 Aquaculture and Fisheries Development Centre, School of Biological, Earth and Environmental Sciences, University College Cork, Cork, Ireland; 3 Marine Institute, Newport, County Mayo, Ireland; University of Rome, Italy

## Abstract

We tested how variation at a gene of adaptive importance, MHC class I (*UBA*), in a wild, endemic *Salmo trutta* population compared to that in both a previously studied non-native *S. trutta* population and a co-habiting *Salmo salar* population (a sister species). High allelic diversity is observed and allelic divergence is much higher than that noted previously for co-habiting *S. salar*. Recombination was found to be important to population-level divergence. The α1 and α2 domains of *UBA* demonstrate ancient lineages but novel lineages are also identified at both domains in this work. We also find examples of recombination between *UBA* and the non-classical locus, *ULA*. Evidence for strong diversifying selection was found at a discrete suite of *S. trutta UBA* amino acid sites. The pattern was found to contrast with that found in re-analysed *UBA* data from an artificially stocked *S. trutta* population.

## Introduction

Genes of adaptive importance are of growing interest to conservation genetics [1]. Major Histocompatibility Complex loci are critical to immune function and highly polymorphic. MHC molecules are loaded with peptides (small fragments of proteins) and transport these to the cell surface. There, the peptide-MHC complex interacts with T cells and, if the peptide is identified as foreign, an immune response is initiated. Variation at MHC affects their ability to bind different types of peptide and is adaptive in helping to resist disease [2]–[5]. Populations which lose this variation [6], [7] may be of conservation concern [1]. Recently, brown trout (*Salmo trutta* L.) have shown promise as a model species for MHC studies. MHC class I showed lower population differentiation than neutral markers across trout populations while variation at class I was maintained in populations isolated above waterfalls where it was lost at neutral markers [8]. Both of these phenomena are expected for a gene under balancing selection. Kin association based on sharing alleles at MHC class I has been demonstrated in the same trout [9]. These studies were based on a MH class I marker and, consequently, it is of clear interest to examine allelic diversity, sequence polymorphism and selection at class I itself in *S. trutta*.

Salmonids possess single expressed or, “classical” Major Histocompatibility Complex class I and II loci [10]–[13]. These loci are unlinked [11], making them exceptional amongst taxa with the “minimal essential MHC” [14]. An antigen processing gene, TAP1, is also on a separate linkage group [15]. MH class I features recombination between ancient and highly divergent α1 and α2 lineages which are 20–100 million years old [12] and may be remnants of distinct loci which have coalesced on a single locus via interlocus exon shuffling [10], [12], [16]–[18].

These ancient lineages demonstrate trans-species polymorphism, an indicator of balancing selection [19], [20]. This may be driven by pathogens [21]–[25], sexual selection [2], [26]–[31]; the influence of recessive deleterious mutations [32] and/or kin selection [9]. Eight class I (*UBA*) α1 lineages and three α2 lineages have been described to date [33]. Interlocus recombination between *UBA* and non-classical loci [16], [34], [35] has not been observed thus far. Recombination at the ∼15kb intron (See Figure S1) between the α1 and α2 domains appears to be a dominant factor in generating novel alleles at *UBA* [11], [12] and was shown to be important to population level divergence in Atlantic salmon, *Salmo salar* L. [36].

Primates show more rapid turnover of alleles at MHC class I than class II with ancient trans-specific lineages observed in the latter. The difference in turnover rate arises from class II proteins binding a broader range of antigens than class I [37]. The opposite pattern is seen in salmonids [12], where it has been attributed to the lack of linkage between loci. However, the same pattern is seen in *Xenopus laevis* MHC class I and class II loci, which are linked [38]. A possibility is that salmonid class I alleles have broader binding capacity than class II. Non-conventional T-Cell Receptor-pMHC binding of “bulged” antigens has been identified in human MHC class I where just a small number of MHC residues are involved in antigen presentation [39]. Hypothetically, this could be important at salmonid class I and these alleles might be able to present a variety of antigens despites shifts in antigenic pressures. A prediction of this theory would be that the pattern of codon level selection would highlight the importance of these key residues.

There is growing emphasis on adaptive loci in population genetics and recent studies of *S. trutta* (employing a MH class I-linked marker), have revealed interesting biological phenomena [8], [9]. Consequently, we seek to supplement these studies and help address key questions in conservation genetics by examining polymorphism at MH class I itself in a wild *S. trutta* population for the first time. Existing data for MH class I from *S. trutta* are from a limited sample size of a non-native introduced strain in the Colorado River, USA [12], which will have been exposed to novel pathogens and may have experienced bottlenecking. How do patterns of allelic diversity, divergence and codon-level selection differ between the wild and artificial stock? A previous study examined MH class I in *S. salar* which share the same Irish river and similar exposure to pathogens over time [36], and here we investigate how the native brown trout compare with these? it was felt that the new data from wild brown trout might also reveal important phylogenetic novelties and help identify whether patterns of selection vary amongst salmonid species.

## Materials and Methods

### Ethics statement

Electrofishing and sampling were carried out under the Certificate of Authorisation for Purposes of the Fisheries Acts 1959–2003, issued to P. McGinnity by the Irish Minister for Communications, Marine and Natural resources. There is no formal ethics committee in the Marine Institute, who were responsible for the capture and killing of the fish. However, the Institute, as an Irish Government agency, has over sixty years of experience working with salmonid fish both cultured and wild and has always taken the upmost care to handle and manage the animals it studies and works with as humanely as possible. Electrofishing was undertaken using standard battery powered 12-volt Safari Research 550E back pack electrofishing equipment (supplied by GFT electrofishing equipment http://www.gft.ie/) for the capture of fish in small streams and rivers. The electrofishing equipment causes the fish to be displaced from it's holding place in the stream into the flow enabling a second person to capture the fish using a handnet. The fish were killed immediately after electrofishing by percussive stunning such that the blow was delivered with sufficient force above or adjacent to the brain in order to render immediate unconsciousness and therefore humane killing of the fish.

### Sampling & *UBA* sequencing

The Srahrevagh River, Co. Mayo, Ireland is a tributary of the Burrishoole River system, where salmonid populations have been extensively studied [8], [9], [36], [40], [41]. As part of these ongoing research efforts, a total of 107 *S. trutta* (1^+^ and older) were sampled from the Srahrevagh on the 15^th^ June 2004 by electro-fishing. Portions of anterior head kidney from all fish were taken under sterile conditions and stored in RNAlater™ (Qiagen Ltd., West Sussex, UK) to prevent RNA degradation. These were transported to the laboratory on ice and stored at −20°C. All individuals were screened for *Sasa-UBA-3UTR*, a microsatellite marker embedded in the 3′ untranslated region of the MHC class I locus [11].

We then selected 28 individuals for *UBA* sequencing. The relationship between the linked microsatellite marker and *UBA* in trout was of interest to a parallel study [41]. To this end, we excluded the small number of fish which were homozygous for the marker (15/107) from otherwise random sub-sampling and included one fish (RW_107) which had a rare marker allele (128). This one individual was not included in the codon by codon selection analysis described below. The exclusion of homozygotes for the marker could have introduced some potential for bias but marker genotypes proved to be unable to predict *UBA* genotypes [41]. Therefore, we concluded that any bias in our sub-sample for *UBA* was minor (See also Table S3).

Head kidney samples were homogenized in lysis buffer [4 M guanidium thiocyanate, 25 mM sodium citrate (pH 7), 0.5% sarkosyl ( = N Lauroyl-sarcosine), 0.1 M β mercaptoethanol], followed by phenol/chloroform extraction. Total RNA was precipitated in ethanol, washed, and dissolved in water. Extracted RNA quality was assessed on a 1% agarose gel and quantified. Working solutions (0.5 mg/ml) of RNA were generated for RT-PCR.

The Superscript™ One-Step RT-PCR with Platinum® *Taq* system (Invitrogen) was used for first strand cDNA synthesis from the RNA isolates and subsequent PCR amplification. The sense primers were: *SatrUBA*F1 5′–TAT TAT CTT GCT GGT GCT GGG AAT–3′; *SatrUBA*F2 5′–TTT CAT CAT TTT GCT CCT GGG AAT–3′) [12]; and the reverse primer was: *SatrUBA*R 5′–GGG TCT TCT GGA GCA GAG ACA–3′; which were designed using available *S. trutta UBA* sequences (Genbank accession numbers AF296374–296383). RT-PCR used the following program: 50°C–30 min; (95°C–2 min, 94°C–30 sec 55°C–30 sec, 72°C–1 min) ×38 cycles; 72°C–7 min. Putative cDNA products were tested on 1% agarose gel.

The ∼600 bp cDNA products were purified on micro bio-spin chromatography columns with 600 µl of Sephacryl S-400 HR matrix. Ligation of the purified cDNA into pGEM®-T Easy Vector and cloning was done as per the manufacturers' instructions with a minor modification (samples were spun down and 850 µl of the supernatant removed to facilitate concentration of the bacterial cells). Some 40 µl of the suspension of bacterial cells was plated out onto LB/ampicillin/IPTG/X-Gal plates and incubated overnight at 37°C. Single colonies were grown in LB broth with ampicillin overnight at 37°C and shaken at 300 rpm. Plasmid DNA was isolated from single colonies using the QIAGEN® QIAprep spin miniprep kit (QIAGEN, Valencia, CA, USA). Both strands of five clones from each of the subsequent PCR amplifications were sequenced using the ABI Prism Bigdye Terminator Cycle Sequencing Ready Reaction kit (Perkin-Elmer, Branchbury, USA) and the T7 and SP6 primers, and analysed on an ABI 377 automated sequencer (Applied Biosystems, Foster City, USA).

### Data preparation

The Sequencher® program was used for contig assembly from ABI® outputs. Novel alleles were identified via comparison with published *Satr-UBA* sequences (Accession Numbers AF296374-AF296383) [12]. Relevant additional salmonid *UBA* data were obtained from Genbank, *S. trutta* (*Satr-UBA*)– Accession Numbers AF296374-83 [12], *S. salar* (*Sasa-UBA*), -AY762572-98 [36]; DQ091795 and DQ091797 [16]; AF504013-17, AF504019-25 and AF 508864 [11] and *O. mykiss* (*Onmy-UBA*), AF091785; AF287483-92; AF296359-73; AF318187-90; AY278451-56; DQ091771-72. The sequences were aligned based on the translated amino acid sequences using ClustalX v1.83 and ClustalW as implemented in MEGA v3.1 [42] with manual editing when necessary. DNA alignments were also generated for the *UBA* α1 and α2 domains, independently. The *S. trutta UBA* allele *Satr*-UBA*0101 (AF296374) was used as a reference sequence for all subsequent analyses. Hereafter, codons cited in the text refer to the translated amino acid sequence of Satr-UBA*0101, with codon 1 (Val1) corresponding to the first codon of the α1 domain of Satr-UBA*0101.

### Basic descriptive statistics

DNASP v4 [43] was used for conducting basic descriptive analysis. Ratios of non-synonymous (d_N_) to synonymous (d_S_) nucleotide substitutions were calculated. These are used in helping to identify the presence of diversifying selection, with ratios of d_N_/d_S_ >1 generally accepted as indicative of selection.

An allelic richness statistic was generated by calculating the number of *UBA* alleles found in 1,000 random samples of ten fish from our total sample of 28 fish, using in-house Python scripts, to provide for direct comparison with the allelic diversity found in the Colorado River brown trout (n = 10, N_A_ = 10) [12].

The program PERMUTE was used to conduct a permutation test for recombination (100,000 permutations in all cases), using the correlation between three measures of linkage disequilibrium (r^2^, D' and G4) and physical distance [44].

### Codon by codon analysis of selection and recombination

OMEGAMAP was used for Bayesian co-estimation of selection (d_N_/d_S_, termed ω hereafter) and recombination (ρ) on MH class I alleles. This analysis excluded sequences from four fish (RW_11F, RW_37F, RW_61M, RW_90M, RW_107F–Table S1) which microsatellite analysis suggested may have arisen from an isolated upstream population [8] to avoid a violation of its assumption of a single population. An alignment consisting of those *Satr*-*UBA* alleles identified from the Srahrevagh River at their frequencies within the sample was constructed for use in OMEGAMAP [44]. The frequency data used in OMEGAMAP are presented in Table S1. Equilibrium codon frequencies were estimated from an alignment of salmonid *UBA*. Reversible-jump MCMC was run twice for each analysis with 250,000 iterations and a burn-in of 25,000 iterations. Details of priors are given in Table S2. The method was found to be robust to the use of alternative priors. Both runs were compared for convergence at several parameters and merged to obtain posterior distributions. A companion program, SUMMARIZE was used for analysis of the OMEGAMAP output. Graphs of data were produced using R scripts provided with OMEGAMAP [45].

### Comparative analysis of stocked Colorado River *S. trutta* using OMEGAMAP

The earliest records of brown trout in Colorado are from the years 1885 and 1886, when state and private hatcheries reported having "English" trout (imported from England). In 1890, the federal hatchery at Leadville began the propagation and distribution of "Von Behr" trout and "Loch Leven" trout. Thus, the ancestry of brown trout now occurring in the headwaters of the Colorado River probably represents a mixture of brown trout from Germany, England, and Scotland (Dr. Robert J. Behnke, Department of Fish, Wildlife, and Conservation Biology, Colorado State University, Fort Collins, Colorado, USA, Pers. Comm.). The *UBA* data from introduced *S. trutta* populations in the Colorado River (USA) [12] allowed the construction of “PAC”-type datasets for analysis in OMEGAMAP using a block model and Prior A, as above. This allowed further comparisons between codon-by-codon selective patterns in *UBA* in populations of different taxa, cohabiting and otherwise. Estimates of ω from the *S. trutta* PAC dataset from the Srahrevagh River were compared with those for the introduced *S. trutta* in the Colorado River. We were interested in the relative strength of selective pressures on a codon-by-codon basis in different populations. OMEGAMAP analyses of three sub-samples of the Srahrevagh data of the same size as that of the Colorado River (n = 10) demonstrated that ω estimates were robust to differences in the size of the data set (data not shown). To test for pairwise differences in the posterior distribution of ω at each codon between the outputs for any two populations, A and B, the 95% Highest Posterior Density interval (a Bayesian analogue of confidence intervals outputted by OMEGAMAP for parameter estimates) for log(ωA/ωB) was calculated at each codon. The hypothesis that ωA = ωB was rejected when the 95% Highest Posterior Density (HPD) interval did not include log(ωA/ωB) = 0.

Descriptive statistics for DNA diversity were again calculated in DNASP.

### Phylogenetic analysis

Neighbor-Net, as implemented in SPLITSTREE v4 [46], works similarly to Neighbor-Joining tree algorithms. Each taxon is initially represented by a single node with iterative agglomeration of neighbouring pairs of nodes into a composite node. However, it differs in that these neighbours are not amalgamated immediately but, rather, this only occurs when a node has been paired up a second time. The three linked nodes are then replaced with two linked nodes and the distance matrix is reduced. By reversing the amalgamation process, the splits given in the Neighbor-Net are produced. These are a circular collection of splits. Graphically, splits are represented by sets of parallel lines separating groups. PROTTEST v1.3 [47] was used to select the best-fit model of protein evolution for overall *Satr*-*UBA* alignments; for α1; and α2 domain alignments, independently. NeighborNet networks were computed with edge weights estimated using ordinary least squares variance and a threshold of 10^−6^ in SPLITSTREE v4. The equal angle algorithm was employed. Maximum likelihood protein distance estimates under the appropriate PROTTEST model were used in generating networks. Bootstrap support with 1000 replicates was provided, but displayed only for the most significant splits for presentation clarity. Networks were generated for *Satr*-*UBA* sequences as a whole and for separate α1 and α2 domain alignments of each, with appropriate *S. salar* and *O. mykiss* outgroups. The models used in each case consisted of the JTT matrix [48] with additional parameters for whole *Satr*-*UBA* (“+I” = 0.127; “+G” = 0.721), α1 (“+G” = 1.163) and α2 (“+G” = 0.386). Where identical α1 or α2 domain “alleles” occurred, a single node was presented. A neighbour-joining tree was also constructed in MEGA v3.1 for salmonid *UBA* amino acid sequences using a JTT matrix with gamma distributed rate variation (+G) of 0.721. Bootstrap support values (1,000 replicates) are presented.

SPLITSTREE can help identify recombination events as incongruities or loops in networks. Specific recombination events within *Satr*-*UBA* sequences, in particular, and salmonid *UBA* in general were analysed in parallel to phylogenetic analysis using SPLITSTREE. Potential events were then examined using MAXCHI in RDP2 [49] and by simple eyeballing of the data using the sequence alignment explorer in MEGA v3.1 [42]. Sequences that have been heavily involved in recombination events, which has been observed in other salmonids [12], or show evidence of intraexon recombination, were noted.

### Interspecific comparisons of selected codons in *UBA* in salmonids

CODEML [50] was used to analyse available *UBA* sequence data from *S. trutta*, *S. salar* and *O. mykiss* because it does not require that the analysis is being carried out on a single population. We predicted that the pattern of selected codons should be conserved amongst these taxa due to the ancient nature of the polymorphism at the locus and possible similarities in the selective pressures over time. Further, any differences which do occur should follow the pattern which might be expected from the established phylogenetic relationships, namely *S. trutta* and *S. salar* should show a more similar pattern of selection than either do with *O. mykiss*.

DNA Maximum Likelihood (DNAML) program version 3.5c [51], as implemented in BioEdit ver 7.0.1 [52], was used to construct maximum likelihood trees for each data set for use in CODEML. CODEML detects positive selection via likelihood-ratio tests between nested probabilistic models [M0 (null), M1a (neutral), M2a (selection), M7 (β) and M8 (β and ω)] of variable ω ratios between codons where the simpler model differs from the more complex model by not allowing for ω>1. Akaike Information Criterion (AIC) statistics were used to test the relative likelihood of models.

### SWISS-MODEL and SPDV DEEPVIEW modelling

The reference *Satr*-*UBA* allele, *Satr*-UBA*0101 was submitted to SWISS-MODEL. The model used was murine MHC I 2bvoA [53] with which *Satr*-UBA*0101 showed 57% similarity. The returned Protein Data Bank files were loaded into the supplied SPDV DEEPVIEW program for three-dimensional visualisation, graphical manipulations, and the plotting of codons under different selective pressures. SPDV DEEPVIEW was used to output files for the rendering software POV-RAY. This produces very high quality graphics of the protein.

## Results

### Descriptive statistics for the Srahrevagh River population

Twenty-one alleles were identified in the Srahrevagh, all of which were novel. The alleles were named *Satr*-UBA*1101-*3101* and Genbank accession numbers AM262749-69 have been allocated to them (Figure S2). Individual genotypes were typical of a single, diploid expressed *Satr*-*UBA* locus except one individual which presented three *Satr*-*UBA* alleles. These alleles did not co-segregate in other individuals as would be expected for a haplotype with tandemly duplicated class I loci. Some 18 of the twenty-eight individuals yielded only one *Satr*-*UBA* allele. This seemed a low level of heterozygosity given the level of allelic diversity observed and, although some form of underdominance may be occurring, it likely reflects preferential amplification of one or other allele given the use of two forward primers. Both problems were noted in previous work on salmonid MHC [12], [54]. The *Satr*-*UBA* alleles were composed of sixteen α1 sequences (14 novel), and nineteen α2 sequences (15 novel). Nucleotide diversity (π) for the Srahrevagh River was 0.260 (see Table 1). Higher divergence and diversity was seen at α1 than α2 but the ratio d_N_/d_S_ is somewhat higher in α2 (Table 1). Both values are around 0.5, much less than 1, implying that the total region is not under diversifying selection using this simple measure.

**Table 1:** Descriptive statistics for *S*atr-*UBA* DNA sequence data from the Srahrevagh River, Co. Mayo.

Nucleotide Diversity	α1	α2	Total
Haplotypes	16	19	21
Sequence length	273	279	552
Sites (excluding gaps)	243	276	519
Polymorphic sites	180	154	334
% Polymorphic sites	65.9%	55.2%	60.5%
Total mutations	292	227	519
Nucleotide diversity (π)	0.32±0.005	0.21±0.005	0.26±0.004
θ per sequence [67] (Ne±SE×10^6^)	50±0.8 (4.2±0.07)	42±0.8 (3.4±0.07)	92±1.1 (3.8±0.05)
Average number of nt differences	78	57	135
Synonymous π (dS)	0.512	0.323	0.413
Non-synonymous π (d_N_)	0.267	0.175	0.218
d_N_/d_S_	0.521	0.542	0.528
Recombination tests			
r^2^	−0.003	−0.063	−0.243***
D'	0.019	−0.030	−0.227***
G4	−0.006	−0.053	−0.215***

Standard errors are presented where relevant. Watterson [67] population mutation rate estimates (θ) are included together with effective population size (N_e_) estimates assuming a mutation rate of 1.1×10^−8^ per site per sequence [68].

PERMUTE [44] found significant evidence for interdomain recombination over the gene as a whole but not for intradomain recombination, when the α1 and α2 domains were considered separately (Table 1).

### Codon by codon analysis of selection and recombination in the Srahrevagh River population

**Figure 1:**
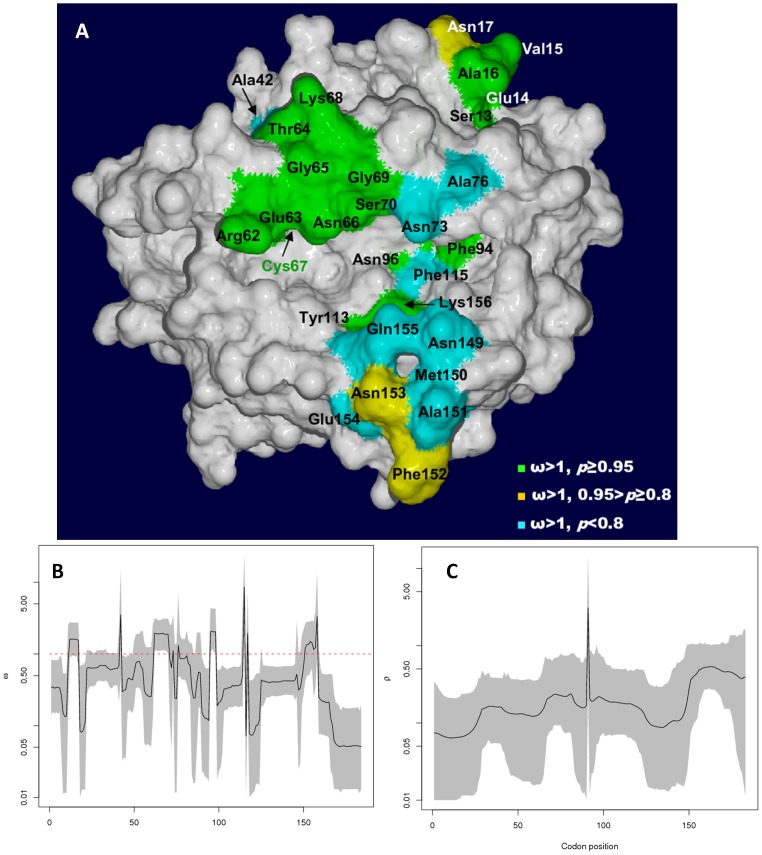
Selection and recombination in the Srahrevagh. A) Model of the peptide binding region of the reference allele, Satr-UBA*0101. Sites under selection are labelled and colour coded according to their degree of statistical support (see key at bottom right of diagram. Higher *p* values indicate stronger statistical support). The sites with the highest ω estimates are Tyr113 (ω = 8.57), Ala42 (3.51), Lys156 (3.38), Phe94 (2.08) and Asn96 (2.05). The Lys156 residue appears to occur between the cleft and the so-called “gatekeeper” residue, Gln155. B, C) Plots of site-by-site mean posterior estimates of ω (B) and ρ (C) for *Satr-UBA* described in this study showing non-correspondence in their pattern of variation. Highest Posterior Density (HPD) 95% confidence intervals are seen in grey about the plot line. In B), the dashed red line indicates ω = 1, values above which indicate selection.

OMEGAMAP showed that codons for 20 amino acid positions were under significant positive selection in *Satr*-*UBA* from the Srahrevagh trout (Figures 1A, 1B, S7). Fourteen of these were in the α1 domain and six were in the α2 domain. Mean ω for the entire *Satr*-*UBA* region was 0.65±0.062 (Table 2), ranging from 0.051 (Asp173)–8.570 (Tyr113). Low background ω rates suggest that most of the *UBA* gene is under purifying selection (Table 2). Evidence for strong positive or diversifying selection was found to occur at a discrete set of codons, (Figure 1A, 1B). Despite more codons being under selection in α1, those under the strongest selection were found within the α2 domain. The ω estimate for Tyr113 was thirteen times the mean ω, and that for Lys156 was five times the mean.

**Table 2:** Summary of OMEGAMAP analyses.

	Srahrevagh	Colorado (*S. trutta)*	Colorado (*O. Mykiss*)
Mean ω	0.65±0.062	0.48±0.022	0.56±0.021
Mean ω (Sites ω≤1)	0.37±0.019	0.41±0.015	0.48±0.009
Mean ω α1	0.76±0.064	0.60±0.031	0.51±0.009
Mean ω α2	0.55±0.106	0.37±0.027	0.60±0.040
Mean ρ	0.22±0.019	0.29±0.008	0.08±0.004
Mean ρ α1	0.14±0.006	0.28±0.010	0.10±0.005
Mean ρ α2	0.26±0.016	0.29±0.013	0.05±0.005

The full output of model parameters including estimates of R, θ, κ and Φ are given in Table S2. Data for an OMEGAMAP analysis of Colorado River *O. mykiss* are also included.

A recombination hotspot was found between codons 91 and 92, where ρ was 3.093 (confidence limits 0.250–14.201), 14 times the mean value of 0.22±0.019 (Table 1, Figure 1C). This marks the position of the large intron II between the exons II and III coding for the α1 and α2 domains, respectively.

No evidence of a correlation was found between ω and ρ co-estimates for positions (Pearson correlation = −0.091, *P* = 0.219), as can be seen in the lack of correspondence in plots of ω and ρ (Figures 1B, 1C). Outside of the important recombination hotspot, selection may be more important in generating new alleles and both factors do not necessarily act on the same codons.

### Comparative analysis of stocked Colorado River *S. trutta* using OMEGAMAP

**Figure 2:**
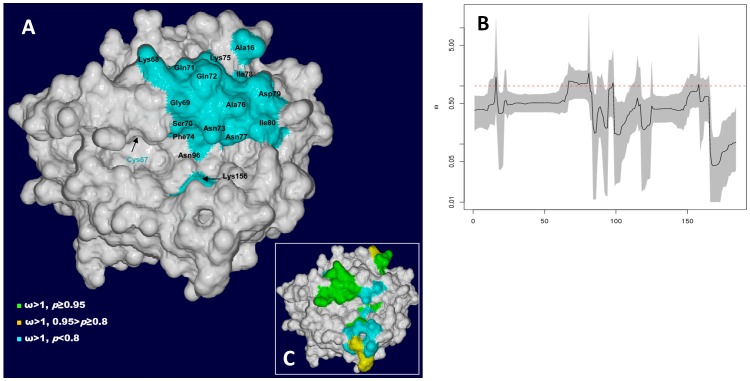
Selection in the Colorado. A) Model showing selected sites in the *UBA* protein for the Colorado River *S. trutta*. For comparison, this information from the Srahrevagh River *S. trutta* population is also provided (inset, right, detail in Figure 1A). Clear differences in the distribution of selected sites in the peptide binding can be seen. B) Plot of ω for the Colorado River *S. trutta*. Highest Posterior Density (HPD) 95% confidence intervals are seen in grey about the plot line and are tight about means in all cases, suggesting confidence in the ω estimates.

Re-analysis of the *S. trutta UBA* data from the Colorado river [12] showed no evidence of significant levels of selection at any amino acid site in the OMEGAMAP analysis of *S. trutta* (Figures 2A, 2B, S7). Mean ω was 0.48±0.022 for *S. trutta* in the Colorado River, which is lower than that found in the Srahrevagh. Mean ρ was slightly higher for *S. trutta* in the Colorado River (0.29±0.008). Curiously, there was no significant evidence for a high ρ estimate at the transition point between the α1 and α2 domains (Table 2).

Comparison of the results from both analyses showed that no codons had significantly higher ω estimates in the Colorado River than in the Srahrevagh population. In contrast, the Srahrevagh had significantly higher ω at codons for **Arg62**-**Gly65** and **Tyr113**; and **Ser12**-**Ala16**; **Arg62**-**Gly69**; **Val93**-**Asn96**; and **Tyr113** than those amino acid positions in the Colorado River. The mean ω_1_/ω_2_ value for the brown trout comparisons with the Colorado River was 1.28±0.103 (SE). The total ω value was not greatly higher (∼20%) suggesting the differences in ω arise at discrete residues or selective foci in the PBR. Higher ω estimates were also found in three sub-samples of the Srahrevagh of n = 10 alleles (data not shown), indicating the pattern is not an artefact of sample size differences.

DNA diversity statistics are presented in Table 3. Divergence levels are somewhat lower than those seen in the Srahrevagh (Table 1), mainly at the α2 domain. However, effective population size estimates (θ) are somewhat higher per sequence in the Colorado River stock than in the Srahrevagh.

**Table 3:** Descriptive statistics for *S*atr-*UBA* DNA sequence data from the Colorado River, USA.

Nucleotide Diversity	α1	α2	Total
Haplotypes	9	9	10
Sequence length	273	279	552
Sites (excluding gaps)	249	276	525
Polymorphic sites	176	133	309
% Polymorphic sites	64.5%	47.7%	56.0%
Total mutations	250	165	415
Nucleotide diversity (π)	0.29±0.017	0.17±0.013	0.23±0.009
θ per sequence (N_e_±SE×10^6^)	62±1.5 (5.2±0.12)	47±1.3 (3.8±0.11)	109±2.0 (4.5±0.08)
Average number of nt differences	72	47	118
Synonymous π (dS)	0.449	0.222	0.333
Non-synonymous π (d_N_)	0.242	0.155	0.196
d_N_/d_S_	0.539	0.698	0.589
Recombination tests			
r2	−0.023	−0.015	−0.313***
D'	0.005	−0.018	−0.033*
G4	0.005	−0.022	−0.030*

Standard errors are presented where relevant. Watterson [67] population mutation rate estimates (θ) are included together with effective population size (N_E_) estimates assuming a mutation rate of 1.1×10^−8^ per site per sequence [68].

### Interspecific comparisons of selected codons in *UBA* in salmonids

**Figure 3:**
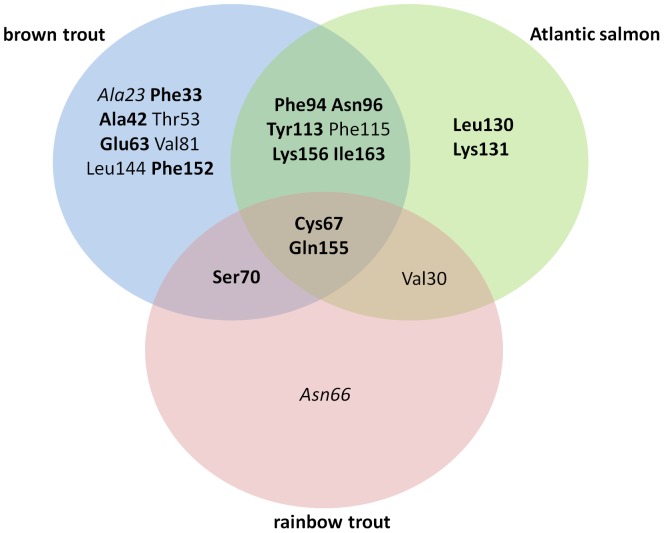
Selected sites in *UBA*. Venn diagram showing all sites under significant selection as identified in conjoint CODEML analysis of the three different taxa labelled. Sites in intersections are under selection in two or more species. Significance levels of selection on residues: p<0.001 (bold), p<0.01 (normal) and p<0.05 (italics).

CODEML models allowing for ω>1 were more likely in likelihood ratio tests, for each taxon, indicating that selection occurs on UBA in each of the three species tested. Codons under selection [M8 (β and ω)] are summarised in Figure 3 with only two codons, Cys67 and Gln155, showed significant evidence of selection in all three salmonid species. Codons in two distinct regions of the protein, Phe94 and Asn96; and Tyr113 and Phe115 were under very highly significant selection in the *Salmo* species but not in *O. mykiss*. More codons are under selection in *S. trutta UBA* than in either of the other two species.

### Phylogenetics

**Figure 4:**
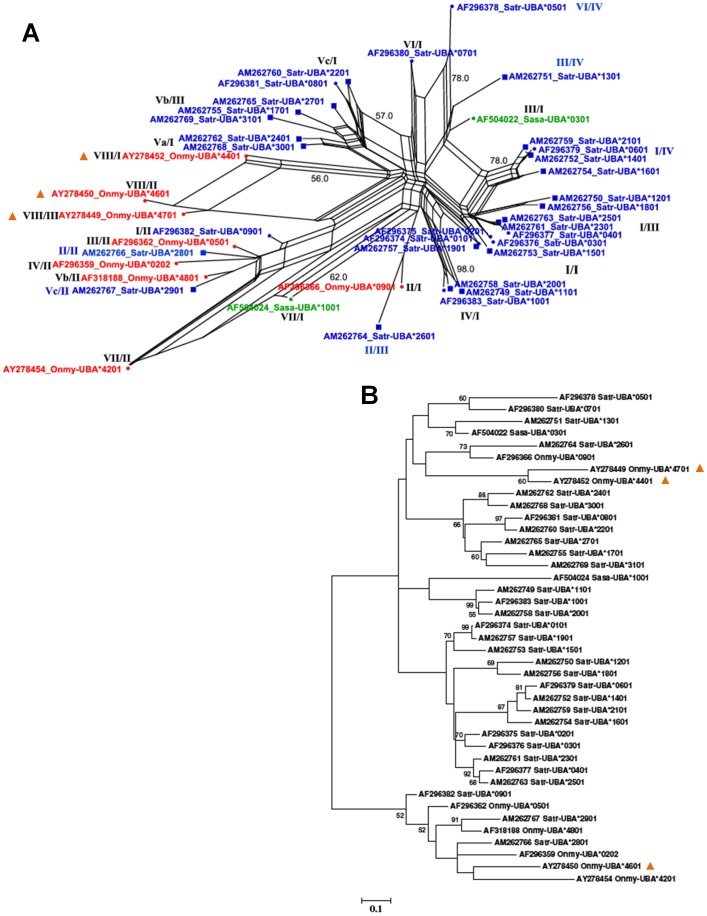
Phylogenetics of *UBA*. A) SPLITSTREE neighbor-net network of *Satr-UBA* alleles (blue) with relevant outgroup sequences from *S. salar* (green) and *O. mykiss* (red). Square nodes indicate the novel alleles identified from the Srahrevagh River, Co. Mayo. Parallel lines indicate splits in the network. Bootstrap support values (1000 replicates) are presented for the most relevant splits in the network. Large loops imply areas of phylogenetic uncertainty or reticulations. The frequency of these in the network implies that recombination is an important factor in the evolution of *Satr*-*UBA*, predominantly between the α1 and α2 domains. Conversely, good bootstrap support for splits involving several closely related *Satr-UBA* alleles is suggestive of conventional radiation by point mutation. Roman numerals (α1/α2) indicate the lineages to which each *Satr-UBA* allele's α1 and α2 sequence belongs (see also Figures 5 and 6). B) Neighbour-joining tree rooted on the midpoint for salmonid UBA amino acid sequences with bootstrap support (1,000 replicates) shown for nodes with 50% support or greater. Nodes in A) and B) highlighted with an orange triangle illustrate how SPLITSTREE is better able to visualise sequences affected by recombination.

SPLITSTREE networks of *Satr*-*UBA* alleles incorporating the twenty one novel alleles described here and relevant salmonid *UBA* outgroups (Figure 4A) demonstrate some large loops, suggesting recombination and/or gene conversion events affecting the alleles connected by those loops. Eleven of 21 (52%) of the *Satr*-*UBA* alleles described here are recombinant alleles. Most of the loops can be explained by recombination at the intron between the exons coding for the α1 and α2 domains of the *Satr*-*UBA* as previously described for Atlantic salmon [12], [36]. Well-supported clades suggestive of conventional radiation by point mutation were also observed (e.g. clades including *Satr*-UBA*1101 and *Satr*-UBA*2301 Figure 4A). A neighbour-joining (NJ) tree of the same data presented for comparison (Figure 4B) shows broad agreement with the SPLITSTREE network. However, alleles which are involved in loops in the network appear to be incorrectly grouped in the NJ tree, e.g. *Onmy*-UBA*4401 (AY278452), *Onmy*-UBA*4701 (AY278449) and *Onmy*-UBA*4601 (AY278450), indicating the utility of the SPLITREE networks for better interpretation of data affected by recombination. Recombinant alleles from the Srahrevagh which are combinations of α1 and α2 lineages which appear to be new to all salmonids are *Satr*-UBA*1201 and *Satr*-UBA*1801 (α1 L_I_/α2 L_III_); Satr-UBA*2601 (α1 L_II_/α2 L_III_); and Satr-UBA*2801 (α1 L_II_/α2 L_II_). Ten of the 22 (45.4%) α1/α2 lineage combinations observed in all salmonid *UBA* data are observed in the single brown trout population from the Srahrevagh.

**Figure 5:**
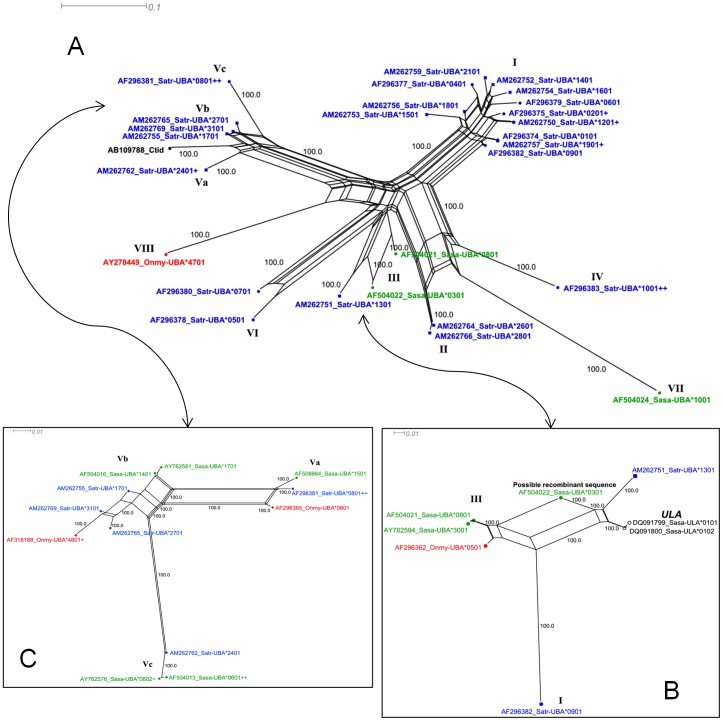
Phylogenetics of the α1 domain. A) *Satr-UBA* α1 sequences (blue) together with relevant outgroup sequences from *S. salar* (green) and *O. mykiss* (red). Novel Srahrevagh River sequences are represented by square nodes. Accession numbers are included in node labels. The number of plus signs after a sequences indicates the number of other *Satr-UBA* alleles which share this sequence in its entirety. α1 lineages are indicated using roman numerals. A *C. idella UBA* is included to highlight the distinct sub-lineages in L_V_, not as an outgroup, and these networks are unrooted. B) Possible α1 intradomain recombination event between typical α1 L_III_ sequences and sequences more similar to Satr-UBA*1301 giving rise to Sasa-UBA*0301. The α1 L_I_ sequence is included as an outgroup. C) α1 L_V_ sequences from *S. trutta*, *S. salar* and *O. mykiss*. Loops are observed in the network, affecting L_Vb_ sequences primarily. Note also in this network the extent of trans-species polymorphism in L_Va_ sequences.

**Figure 6:**
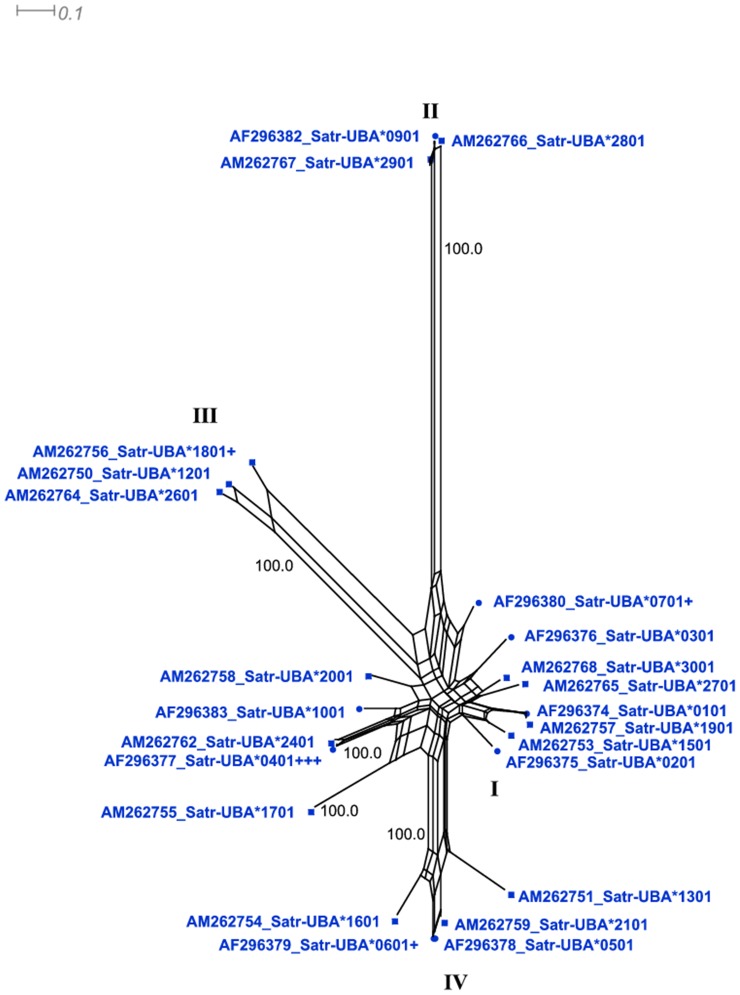
Phylogenetics of the α2 domain. A) *Satr-UBA* α2 sequences with novel sequences described in this work represented by square nodes. The number of plus signs after a sequence indicates the number of other *Satr-UBA* alleles which share this sequence in its entirety and, therefore, are sequences which are likely to have been involved in recombination. Known α2 lineages are indicated using roman numerals. Note that a novel α2 lineage, L_IV_, unique to *S. trutta*, which appears to have originated more recently from the α2 L_I_ lineage, is well supported with the additional data described in this work. The shape of the overall tree is distinct from that of α1 with fewer well-supported lineages and with evidence of extensive radiation within the ‘majority’ α2 L_I_ lineage.

Several groups of alleles share the same α1 DNA sequence [i.e. Satr-UBA*0801/*2201/*2901 have identical α1, Satr-UBA*1001/*1101/*2001, Satr-UBA*0201/0301, Satr-UBA*1201/*2501, Satr-UBA*1901/*2301 and Satr-UBA*2401/*3001 (Figure 5A)], or the same α2 sequences [Satr-UBA*0401/*0801/*2301/*2501, Satr-UBA*0601/*1401 and Satr-UBA*1801/*3101 (underlined alleles are those from the Colorado River) (Figure 6)]. It is clear from these data that recombination is a major factor in population level divergence in brown trout, as found to be the case in *S. salar* [36].

### Phylogenetics of α1 domain sequences

This study extends the number of ancient salmonid α1 lineages recorded in *S. trutta*. The α1 network is broadly tree-like, but features a few loops, suggesting intra-domain recombination between deeply diverged and ancient α1 lineages can also occur (Figure 5A). Two loops warrant additional discussion. The relationship of the α1 sequence of Satr-UBA*1301 to previously described α1 L_III_ sequences is marked by a loop in the network (Figure 5A). When *Sasa*-UBA*0301 was removed from the network (analysis not shown), the Satr-UBA*1301 did not cluster with α1 L_III_ sequences. Closer examination shows *Satr*-UBA*1301 and Sasa-UBA*0301 have been involved in separate intradomain recombination events which involve a sequence shared with the non-classical locus, *ULA*, which is unique to *Salmo spp* (Figures 5B, S4A). The *ULA* locus is on the same linkage group as *UBA* in *S. salar* [16]. These data, together with the fact that none of the reported non-classical loci [16] appear to be related to this allele, suggest that Satr-UBA*1301 is the first representative of a well-supported and novel, ninth α1 lineage at this locus in salmonids. The additional *Satr-UBA* data provided by these novel alleles also reveal additional sub-lineages within α1 L_V_, termed L_Va_, L_Vb_ and L_Vc_ (Figure 5A, 5C). Sub-lineages L_Va_ and L_Vc_ are well-supported and feature characteristic sequence motifs shared amongst salmonids (Figure 5C, S4B). Sub-lineage L_Vb_ is poorly supported and appears to have been generated by multiple reticulations involving alleles from L_Va_ and L_Vc_.

The region downstream of the recombination break point in Satr-UBA*1301 is marked by an amino acid motif between residues Pro59 and Ile66 (PDYWERETQI) which appears to be unique amongst salmonid *UBA* (see Figure S3). This region contains two sites, Tyr59 (conserved) and Glu63 (variable) which form “Pocket A” of the peptide binding cleft with Tyr171, on α2. A BlastP search found the identical amino acid motif in a shark (*Triakis scyllium*) and the Pallid Atlantic Forest Rat (*Delomys sublineatus*) although the differences at the nucleotide level over the same region were 20% and 17%, respectively. Similarly, human HLA-B*4413 differs by a single amino acid from the trout amino sequence but is 23.3% different in its nucleotide sequence. The shark, rodent and human alleles differ from Satr-UBA*1301 by 41% (55% nt), 52% (70% nt) and 56% (71% nt), respectively over the remainder of their amino acid sequences, suggesting some form of convergence in these MHC alleles in taxa separated by over 400 million years [55].

Separate examination of the phylogeny of α1 L_I_ (which contains half of all the salmonid α1 alleles described in Figure S3) shows parts are tree-like and typical of evolution of sequences by simple point mutation, for example, the sub-lineage of *S. salar* alleles including Sasa-UBA*0902 (Figure S8).. However, extensive trans-specific polymorphism within this lineage occurs between the *Salmo* species and with *O. mykiss*. The analysis also highlights a divergent *S. trutta* clade including *Satr*-UBA*0201/0301; *Satr*-UBA*0401; *Satr*-UBA*0601; *Satr*-UBA*1201; *Satr*-UBA*1401; *Satr*-UBA*1601 and *Satr*-UBA*2101 which is characterised by large loops indicating reticulate evolution within this species.

### Phylogenetics of α2 domain sequences

The phylogeny of α2 displays four distinct allelic lineages, three of which are already known in salmonids, but the fourth is novel and unique to brown trout (Figure 6). This study also extends the diversity of α2 lineages recorded in *S. trutta*. Divergence between α2 allelic lineages is far greater than that between α1 alleles. The distinct “majority” type α2 L_I_ lineage (containing two thirds of all the α2 alleles described in Figure S5), and the other two other highly diverged and ancient lineages α2 L_II_ and α2 L_III_, have been maintained in all salmonids. *S. trutta* α2 sequences Satr-UBA*0501, Satr-UBA*1301, Satr-UBA*1401, Satr-UBA*1601 and Satr-UBA*2101 form a divergent, monophyletic and well supported, novel clade, designated L_IV_. The substitution of a hydrophobic valine or methionine residue at Gln95, otherwise conserved across diverse taxa, differentiates this clade from others. This residue is also conserved in non-classical salmonid loci such as *UFA*, *UGA* and *UEA* but not in the *Salmo* specific *ULA* where another hydrophobic residue, leucine, is found. The two positions adjacent to Gln95 are known to be important for peptide binding and were under significant diversifying selection in our OMEGAMAP and CODEML analyses. The α2 Satr-UBA*1301 sequence, part of this new lineage, has an interesting substitution of the positively charged histidine at Gln114. Gln114 is ordinarily conserved across diverse taxa (except the zebra fish *Danio rerio* where it is replaced by negatively charged glutamic acid) and is important to CD8 and β-metaglobulin interactions [56]. This position borders the selection hotspot identified in this work at Tyr113.

Trans-specific polymorphism is pronounced in α2 L_II_ (and to a lesser extent α2 L_III_) where α2 alleles found in *O. mykiss* (e.g. Onmy-UBA*0202) and Satr-UBA*2801 and Satr-UBA*2901 have very similar amino acid sequences. Notably, the entire diversity of salmonid α2 is captured by *S. trutta* (Figure 6) and, indeed, all lineages described were identified in the Srahrevagh brown trout population. In contrast, while α2 L_I_ is clearly very old and exhibits trans-specific polymorphism, there is more evidence of species-specific diversification, including the large number of *S. salar* sequences (Figure S9).

## Discussion

The first MH class I *Satr-UBA* data described from wild, endemic *S. trutta* have revealed a high diversity of alleles within a single population, new allelic lineages in both α1 and α2 domains, strong selection at discrete codons in the locus and the importance of recombination to population level divergence. These data permit new insights into the evolution of MH class I in salmonids, a locus of considerable importance in adapting to novel ecological challenges.

The identification of twenty-one novel alleles, from twenty-eight individual fish demonstrated the high allelic diversity in the Srahrevagh *S. trutta* population. Allelic richness (10.2) was very similar to that in the Colorado River *S. trutta*, N_A_ = 10, (and *O. mykiss*, N_A_ = 10) [12], and the number of alleles was identical to that in *S. salar* (N_A_ = 21) taken from four populations (including the Burrishoole) in the same area of Ireland [36]. However, the alleles in the wild *S. trutta* were more divergent (π = 0.260) than those in *S. salar* (π = 0.184) [36].

No MH class I allele was shared with the only *S. trutta* previously studied, from the Colorado River, although α1 and α2 sequences were shared. This mirrors the situation previously identified in *S. salar* populations [36] and highlights the role of recombination in driving rapid population level divergence at this locus in both *Salmo* species. Contrary to the findings in *S. salar*, however, there was no clear evidence of an interplay of selection and recombination on the same sites. We have also identified clear examples of recombination occurring between lineages at both α1 and α2 and with a non-classical locus, *ULA*. What factors provide for novel recombinant alleles to be functional and readily fixed at the population level?

Recombinant alleles may be more divergent, easier to behaviourally detect [57] (and thus favoured) in sexual selection [29] or kin association (demonstrated to occur in the Srahrevagh *S. trutta* population) [9]. Recombinant alleles are also likely to result in proteins with a radically altered peptide binding region, which may give rise to a divergent allele advantage [58]–[61]. If this is true, the more divergent suite of MH class I alleles found in *S. trutta* than in co-habiting *S. salar* should result in superior ability to detect pathogens, a possibility which could be addressed in pathogen challenge experiments. To extend this point further, in terms of adaptive variation, are populations with more divergent MHC alleles fitter?

However, there may not be an advantage to divergent alleles, as there is evidence for convergent evolution in MHC binding specificities [62]–[65], with human class I classified into as few as nine “supertypes”, defined by overlapping peptide-binding motifs. In short, alleles which appear very different could be functionally similar. The advantage of divergent recombinant alleles to pathogen detection could also be negated by the fact that an important antigen processing gene (TAP1) is located on a separate chromosome to MH class I in salmonids [15], requiring that both proteins evolve to an ‘average best fit’ independently. In that case, the antigen processing genes may not be well adapted to the presentation of different types of peptides to novel divergent *UBA* alleles.

The extent of recombination in salmonid MH class I and this separation of the antigen processing genes imply that antigen presentation in salmonids is extraordinarily plastic. The discrete pattern of selection at class I may also be of note. The stocked *S. trutta* in the Colorado River have retained high allelic diversity but appear to lack variation (Figure 2) at two selective foci identified in the Srahrevagh (Figure 1B), Phe94-Asn96 and Tyr113 (and in CODEML analysis for both *Salmo* species), which occur at the base of the peptide-binding cleft. These would seem important to antigen binding (Figure 1A) and the relative lack of variation in the Colorado population is curious. Interestingly, the stocked *O. mykiss* in the Colorado River show a similar pattern of selected codons to the stocked *S. trutta* (Figures S6A, S6B, S6C).

Additionally, Gln155 is one of only two amino acids positions found to be under strong selection across all salmonid taxa. However, this amino acid position is conserved in human class I and is known to be critical to class I restricted T cell recognition [39]. Gln155 is important to a newly-identified form of antigen presentation in HLA wherein longer peptides are bound bulged out of the peptide binding region (PBR) [39]. Direct interactions between the antigen and the T cell receptor dominate this form of binding, most MHC amino acids are not involved and the shape of the PBR is not likely to be a critical factor. We speculate here that this form of binding may be a feature of salmonid class I molecules. This would help explain how recombination between divergent α1 and α2 allelic lineages can freely occur. This hypothesis could be tested in future studies which identify the nature of antigens bound by different salmonid MH class I alleles.

## Supporting Information

Figure S1 **Salmonid** ***UBA*** **structure.** Relevant structure of the salmonid UBA gene (after [66]) and based on the rainbow trout allele AF296362_Onmy-UBA*0501. Intron-exon organisation is shown with sizes for the relevant exons and introns in nucleotide base pairs given in parentheses. Note the large size of intron II between exons coding for the α1 and α2 domains.
(TIF)

Figure S2 **Novel** ***Satr-UBA*** **alleles.** Amino acid alignment of novel *Satr-UBA* alleles described in this work. Accession numbers are included in each allele name. Sequences from the α1 domain (top) and α2 domain (bottom) are displayed together with the respective lengths of each sequence.
(TIF)

Figure S3 **α1 sequence alignments.** A) Representative salmonid *UBA* α1 domain amino acid sequence alignments capturing the diversity of variation within α1 lineages (roman numerals) and between lineages. We include Satr-UBA*1301 with α1 LIII sequences. When sites which were found to be under selection in OMEGAMAP were considered, it is noted that these fall into two categories, sites which are highly variable between lineages and sites which are highly variable both between and within particular lineages.
(TIF)

Figure S4 **Alignments highlighting recombination in α1 lineages.** A) Nucleotide sequences for Satr-UBA*1301, Sasa-UBA*0301, Sasa-UBA*0801, Sasa-ULA*0102 and an α1 L_I_ sequence, the reference sequence, Satr-UBA*0101. Note that Satr-UBA*1301, Sasa-UBA*0301 and Sasa-ULA*0102 have very similar nt sequences between positions 1 and ∼136 whereupon Sasa-UBA*0301 is observed to abruptly demonstrate greater similarity to a typical α1 L_III_ sequence, Sasa-UBA*0301. Satr-UBA*1301 sequence similarity to the *ULA* sequence persists slightly longer but thereafter large numbers of nt differences are observed. This pattern is typical of recombination or gene conversion events occurring within the α1 domain. B) Amino acid alignments of α1 L_v_ lineages. Note the high degree of similarity between sequences from different species indicating that trans-species polymorphism is extensive in α1 L_v_. Note that sequences in lineage L_Vb_ are more similar to sequences of L_Vc_ between aa positions 1-28 but more similar to L_Va_ sequences in the remainder of the sequence. This pattern might be explained by an ancient recombination event (or events) between L_Va_ and L_Vc_ sequences giving rise the poorly supported L_Vb_ clade. Notably, when α1 L_Vb_ sequences are removed from SPLITSTREE networks (data not shown), L_Va_ and L_Vc_ sequences appear as distinct α1 lineages although sharing a more recent common ancestor than any other pair of lineages in the network. This suggests both that intradomain recombination between lineages is possible but also that it is more feasible between more closely related lineages.
(TIF)

Figure S5 **Alignment of representative salmonid** ***UBA*** **α2 domain amino acid sequence alignments showing the diversity of variation within α2 lineages (roman numerals) and between lineages.** Sites found to be under selection in OMEGAMAP fall into two categories, sites which are highly variable between lineages and sites which are highly variable both between and within particular lineages. A notable feature of α2 diversity is the extensive and diffuse polymorphism within α2 L_I_. In contrast, a remarkable degree of conservation is observed within other α2 lineages. This may point to differences in selective pressures in different α2 lineages.
(TIF)

Figure S6 A) Model showing selected sites in the *UBA* protein for the Colorado River introduced populations of *S. trutta* population (top) and in the Colorado River *O. mykiss* population (bottom). For comparison, this information from the Srahrevagh River *S. trutta* population is also provided (inset, right, detail in Figure 1A). Clear differences in the distribution of selected sites in the peptide binding can be seen. B, C) Comparative plots of ω for the Colorado River *S. trutta* (B) and *O. mykiss* (C) populations. The pattern observed in the *O. mykiss* population is remarkably flat outside distinct diversifying selection foci at Ser70 and between Asn149 and Ile163. Highest Posterior Density (HPD) 95% confidence intervals are seen in grey about the plot line and are tight about means in all cases, suggesting confidence in the ω estimates.
(TIF)

Figure S7 **Selected sites in UBA.** Venn diagrams of sites under selection identified in independent OMEGAMAP analyses of the three individual populations labelled. Significance levels of selection on residues: p<0.001 (bold), p<0.01 (normal) and p<0.05 (italics).
(TIF)

Figure S8 **Phylogenetics of α1 Lineage I.** α1 L_I_ Large loops are observed in the network, particularly affecting *Satr-UBA* sequences, indicating recombination events. Other parts of the network are more treelike, suggesting a stronger role for point mutation. Each salmonid species demonstrates some species-specific diversification but trans-species polymorphism is observed even within this most diverse of α1 lineages.
(TIF)

Figure S9 **Phylogenetics of α1 Lineage I.** The α2 L_I_ network is typified by stellate radiation although incongruities may imply gene conversion, recombination or convergence also occurs. Trans-species polymorphism is observed although no sequences demonstrate a high degree of similarity. In other parts of the network, species-specific diversification is extensive, particularly for *S. salar* sequences.
(TIF)

Table S1 **Srahrevagh UBA allele frequency data.**
(DOCX)

Table S2 **OMEGAMAP prior distribution parameter sets.** Details of prior distribution sets. Prior A was used for analyses.
(DOCX)

Table S3 **Entropy statistics.** This table includes details of codon by codon entropy values for each population. We also include OMEGAMAP estimates and CODEML sites under selection for comparison.
(XLSX)

## References

[1] Gebremedhin B, Ficetola GF, Naderi S, Rezaei HR, Maudet C, et al (2009) Frontiers in identifying conservation units: from neutral markers to adaptive genetic variation. Animal Conservation 12: 107–109.

[2] Bernatchez L, Landry C (2003) MHC studies in nonmodel vertebrates: what have we learned about natural selection in 15 years? Journal of Evolutionary Biology 16: 363–377. 14635837 10.1046/j.1420-9101.2003.00531.x

[3] Muirhead CA (2001) Consequences of population structure on genes under balancing selection. Evolution 55: 1532–1541. 11580013 10.1111/j.0014-3820.2001.tb00673.x

[4] Kurtz J, Kalbe M, Aeschlimann PB, Haberli MA, Wegner KM, et al (2004) Major histocompatibility complex diversity influences parasite resistance and innate immunity in sticklebacks. Proceedings of the Royal Society B: Biological Sciences 271: 197–204. 15058398 10.1098/rspb.2003.2567 PMC1691569

[5] O'Brien SJ, Evermann JF (1988) Interactive influence of infectious disease and genetic diversity in natural populations. Trends in Ecology & Evolution 3: 254–259. 21227241 10.1016/0169-5347(88)90058-4 PMC7134056

[6] Miller HC, Lambert DM (2004) Genetic drift outweighs balancing selection in shaping post-bottleneck major histocompatibility complex variation in New Zealand robins (Petroicidae). Molecular Ecology 13: 3709–3721. 15548285 10.1111/j.1365-294X.2004.02368.x

[7] Weber DS, Stewart BS, Schienman J, Lehman N (2004) Major histocompatibility complex variation at three class II loci in the northern elephant seal. Molecular Ecology 13: 711–718. 14871373 10.1111/j.1365-294x.2004.02095.x

[8] O'Farrell B, Dennis C, Benzie JA, McGinnity P, Carlsson J, et al (2012) Balancing selection on MHC class I in wild brown trout Salmo trutta. Journal of Fish Biology 81: 1357–1374. 22957875 10.1111/j.1095-8649.2012.03421.x

[9] O'Farrell B, Benzie JAH, McGinnity P, Carlsson J, Eyto Ed, et al (2012) MHC-mediated spatial distribution in brown trout (*Salmo trutta*) fry. Heredity 108: 403–409. 21934705 10.1038/hdy.2011.87 PMC3313050

[10] Aoyagi K, Dijkstra JM, Xia C, Denda I, Ototake M, et al (2002) Classical MHC class I genes composed of highly divergent sequence lineages share a single locus in rainbow trout (*Oncorhynchus mykiss*). J Immunol 168: 260–273. 11751970 10.4049/jimmunol.168.1.260

[11] Grimholt U, Drablos F, Jorgensen SM, Hoyheim B, Stet RJM (2002) The major histocompatibility class I locus in Atlantic salmon (*Salmo salar* L.): polymorphism, linkage analysis and protein modelling. Immunogenetics 54: 570–581. 12439620 10.1007/s00251-002-0499-8

[12] Shum BP, Guethlein L, Flodin LR, Adkison MA, Hedrick RP, et al (2001) Modes of salmonid MHC class I and II evolution differ from the primate paradigm. Journal of Immunology 166: 3297–3308. 10.4049/jimmunol.166.5.3297 11207285

[13] Stet RJM, de Vries B, Mudde K, Hermsen T, van Heerwaarden J, et al (2002) Unique haplotypes of co-segregating major histocompatibility class II A and class II B alleles in Atlantic salmon (*Salmo salar*) give rise to diverse class II genotypes. Immunogenetics 54: 320–331. 12185536 10.1007/s00251-002-0477-1

[14] Kaufman J (1999) Co-evolving genes in MHC haplotypes: the "rule" for nonmammalian vertebrates? Immunogenetics 50: 228–236. 10602883 10.1007/s002510050597

[15] Phillips RB, Zimmerman A, Noakes MA, Palti Y, Morasch MRW, et al (2003) Physical and genetic mapping of the rainbow trout major histocompatibility regions: evidence for duplication of the class I region. Immunogenetics 55: 561–569. 14566436 10.1007/s00251-003-0615-4

[16] Miller KM, Li S, Ming TJ, Kaukinen KH, Schulze AD (2006) The salmonid MHC class I: more ancient loci uncovered. Immunogenetics 58: 571–589. 16794819 10.1007/s00251-006-0125-2

[17] Hansen JD, Strassburger P, Du Pasquier L (1996) Conservation of an alpha 2 domain within the teleostean world, MHC class I from the rainbow trout *Oncorhynchus mykiss* . Developmental and Comparative Immunology 20: 417–425. 9040984 10.1016/s0145-305x(96)00030-4

[18] Xia C, Kiryu I, Dijkstra JM, Azuma T, Nakanishi T, et al (2002) Differences in MHC class I genes between strains of rainbow trout (Oncorhynchus mykiss). Fish Shellfish Immunol 12: 287–301. 12049167 10.1006/fsim.2001.0371

[19] Garrigan D, Hedrick PW (2003) Perspective: Detecting adaptive molecular polymorphism: Lessons from the MHC. Evolution 57: 1707–1722. 14503614 10.1111/j.0014-3820.2003.tb00580.x

[20] Klein J, Sato A, Nagl S, O'Huigin C (1998) Molecular trans-species polymorphism. Annual Review of Ecology and Systematics 29: 1–21.

[21] Doherty P, Zinkernagel R (1975) Enhanced immunological surveillance in mice heterozygous at the H-2 gene complex. Nature 256: 50–52. 1079575 10.1038/256050a0

[22] Hughes AL, Nei M (1988) Pattern of nucleotide substitution at major histocompatibility complex class I loci reveals overdominant selection. Nature 335: 167–170. 3412472 10.1038/335167a0

[23] Clarke B, Kirby D (1966) Maintenance of histocompatibility polymorphism. Nature 211: 999–1000. 6007869 10.1038/211999a0

[24] Slade RW, McCallum HI (1992) Overdominant vs. frequency-dependent selection at MHC loci. Genetics 132: 861–864. 1468635 10.1093/genetics/132.3.861 PMC1205221

[25] Spurgin LG, Richardson DS (2010) How pathogens drive genetic diversity: MHC, mechanisms and misunderstandings. Proceedings of the Royal Society of London Series B-Biological Sciences 277: 979–988. 10.1098/rspb.2009.2084 PMC2842774 20071384

[26] Hamilton W, Zuk M (1982) Heritable true fitness and bright birds: a role for parasites? Science 218: 384–387. 7123238 10.1126/science.7123238

[27] Trivers RL (1972) Parental investment and sexual selection. In: Campbell, BG, editors. Chicago: Aldine. pp. 136–179.

[28] Consuegra S, Garcia de Leaniz C (2008) MHC-mediated mate choice increases parasite resistance in salmon. Proceedings of the Royal Society of London Series B, Biological Sciences 275: 1397–1403. 18364312 10.1098/rspb.2008.0066 PMC2602703

[29] Landry C, Garant D, Duchesne P, Bernatchez L (2001) 'Good genes as heterozygosity': the major histocompatibility complex and mate choice in Atlantic salmon (*Salmo salar*). Proceedings of the Royal Society of London Series B-Biological Sciences 268: 1279–1285. 10.1098/rspb.2001.1659 PMC1088738 11410155

[30] Neff B, Garner S, Heath J, Heath D (2008) The MHC and non-random mating in a captive population of Chinook salmon. Heredity 101: 175–185. 18506203 10.1038/hdy.2008.43

[31] Pitcher TE, Neff BD (2006) MHC class IIB alleles contribute to both additive and nonadditive genetic effects on survival in Chinook salmon. Molecular Ecology 15: 2357–2365. 16842411 10.1111/j.1365-294X.2006.02942.x

[32] Van Oosterhout C (2009) A new theory of MHC evolution: beyond selection on the immune genes. Proceedings of the Royal Society B-Biological Sciences 276: 657–665. 10.1098/rspb.2008.1299 PMC2660941 18986972

[33] Kiryu I, Dijkstra JM, Sarder RI, Fujiwara A, Yoshiura Y, et al (2005) New MHC class Ia domain lineages in rainbow trout (*Oncorhynchus mykiss*) which are shared with other fish species. Fish & Shellfish Immunology 18: 243–254. 15519543 10.1016/j.fsi.2004.07.007

[34] Shum BP, Rajalingam R, Magor KE, Azumi K, Carr WH, et al (1999) A divergent non-classical class I gene conserved in salmonids. Immunogenetics 49: 479–490. 10380691 10.1007/s002510050524

[35] Shum BP, Mason PM, Magor KE, Flodin LR, Stet RJ, et al (2002) Structures of two major histocompatibility complex class I genes of the rainbow trout (*Oncorhynchus mykiss*). Immunogenetics 54: 193–199. 12073148 10.1007/s00251-002-0450-z

[36] Consuegra S, Megens HJ, Schaschl H, Leon K, Stet RJM, et al (2005) Rapid evolution of the MH class I locus results in different allelic compositions in recently diverged populations of Atlantic salmon. Molecular Biology and Evolution 22: 1095–1106. 15689529 10.1093/molbev/msi096

[37] Go Y, Satta Y, Kawamoto Y, Rakotoarisoa G, Randrianjafy A, et al (2003) Frequent segmental sequence exchanges and rapid gene duplication characterise the MHC class I genes in lemurs. Immunogenetics 55: 450–461. 14530885 10.1007/s00251-003-0613-6

[38] Bos DH, Waldman B (2006) Evolution by recombination and transspecies polymorphism in the MHC class I gene of *Xenopus laevis* . Molecular Biology and Evolution 23: 137–143. 16162865 10.1093/molbev/msj016

[39] Tynan F, Burrows S, Buckle A, Clements C, Borg N, et al (2005) T cell receptor recognition of a 'super-bulged' major histocompatibility complex class I-bound peptide. Nature Immunology 6: 1114–1122. 16186824 10.1038/ni1257

[40] Consuegra S, Megens HJ, Leon K, Stet RJM, Jordan WC (2005) Patterns of variability at the major histocompatibility class II alpha locus in Atlantic salmon contrast with those at the class I locus. Immunogenetics 57: 16–24. 15726347 10.1007/s00251-004-0765-z

[41] Coughlan J, McGinnity P, O'Farrell B, Dillane E, Diserud O, et al (2006) Temporal variation in an immune response gene (MHC I) in anadromous *Salmo trutta* in an Irish river before and during aquaculture activities. ICES Journal of Marine Science: Journal du Conseil 63: 1248–1255.

[42] Kumar S, Tamura K, Nei M (2004) MEGA3: Integrated software for Molecular Evolutionary Genetics Analysis and sequence alignment. Briefings in Bioinformatics 5: 150–163. 15260895 10.1093/bib/5.2.150

[43] Rozas J, Sanchez-DelBarrio J, Messeguer X, Rozas R (2003) DnaSP, DNA polymorphism analyses by the coalescent and other methods. Bioinformatics 19: 2496–2497. 14668244 10.1093/bioinformatics/btg359

[44] Wilson DJ, McVean G (2006) Estimating diversifying selection and functional constraint in the presence of recombination. Genetics 172: 1411–1425. 16387887 10.1534/genetics.105.044917 PMC1456295

[45] R Development Core Team (2008) R: A language and environment for statistical computing. R Foundation for Statistical Computing,Vienna, Austria. Available: http://www.R-project.org. Accessed: 2013, April 10.

[46] Huson D, Bryant D (2006) Application of Phylogenetic Networks in Evolutionary Studies. Molecular Biology and Evolution 23: 254–267. 16221896 10.1093/molbev/msj030

[47] Abascal F, Zardoya R, Posada D (2005) ProtTest: selection of best-fit models of protein evolution. Bioinformatics 21: 2104–2105. 15647292 10.1093/bioinformatics/bti263

[48] Jones DT, Taylor WR, Thornton JM (1992) The Rapid Generation of Mutation Data Matrices from Protein Sequences. Computer Applications in the Biosciences 8: 275–282. 1633570 10.1093/bioinformatics/8.3.275

[49] Martin DP, Williamson C, Posada D (2005) RDP2: recombination detection and analysis from sequence alignments. Bioinformatics 21: 260–262. 15377507 10.1093/bioinformatics/bth490

[50] Yang Z (1997) PAML: a program package for phylogenetic analysis by maximum likelihood. Computer Applications in the Biosciences 13: 555–556. 9367129 10.1093/bioinformatics/13.5.555

[51] Felsenstein J (1993) PHYLIP (Phylogeny Inference Package) Version 3.5c., version Seattle: University of Washington.

[52] Hall T (1999) BioEdit: a user-friendly biological sequence alignment editor and analysis program for Windows 95/98/NT. Nucleic Acids Symposium Series 41: 95–98.

[53] Stewart-Jones GBE, Gillespie G, Overton IM, Kaul R, Roche P, et al (2005) Structures of three HIV-1 HLA-B*5703-peptide complexes and identification of related HLAs potentially associated with long-term nonprogression. Journal of Immunology 175: 2459–2468. 10.4049/jimmunol.175.4.2459 16081817

[54] Hansen JD, Strassburger P, Thorgaard GH, Young WP, Du Pasquier L (1999) Expression, linkage, and polymorphism of MHC-related genes in rainbow trout, *Oncorhynchus mykiss* . J Immunol 163: 774–786. 10395670

[55] Zhu M, Yu XB, Janvier P (1999) A primitive fossil fish sheds light on the origin of bony fishes. Nature 397: 607–610.

[56] Collins EJ, Riddle DS (2008) TCR-MHC docking orientation: natural selection, or thymic selection? Immunol Res 41: 267–294. 18726714 10.1007/s12026-008-8040-2

[57] Carroll L, Penn D, Potts W (2002) Discrimination of MHC-derived odors by untrained mice is consistent with divergence in peptide-binding region residues. Proceedings of the National Academy of Sciences of the United States of America 99: 2187–2192. 11842193 10.1073/pnas.042244899 PMC122340

[58] Miller KM, Kaukinen KH, Beacham TD, Withler RE (2001) Geographic heterogeneity in natural selection on a MHC locus in sockeye salmon. Genetica 111: 237–257. 11841169 10.1023/a:1013716020351

[59] Lenz TL (2011) Computational prediction of MHC II-antigen binding supports divergent allele advantage and explains trans-species polymorphism. Evolution 65: 2380–2390. 21790583 10.1111/j.1558-5646.2011.01288.x

[60] Takahata N, Satta Y (1998) Selection, convergence, and intragenic recombination in HLA diversity. Genetica 102–103: 157–169. 9720277

[61] Wakeland E, Boehme S, She J (1990) Ancestral polymorphisms of MHC class II genes: divergent allele advantage. Immunological Research 9: 115–122. 10.1007/BF02918202 2189934

[62] Yeager M, Kumar S, Hughes A (1997) Sequence convergence in the peptide-binding region of primate and rodent MHC class Ib molecules. Molecular Biology and Evolution 14: 1035–1041. 9335143 10.1093/oxfordjournals.molbev.a025709

[63] Yeager M, Hughes AL (1999) Evolution of the mammalian MHC: natural selection, recombination, and convergent evolution. Immunological Reviews 167: 45–58. 10319250 10.1111/j.1600-065x.1999.tb01381.x

[64] Sette A, Sidney J, Livingston B, Dzuris J, Crimi C, et al (2003) Class I molecules with similar peptide-binding specificities are the result of both common ancestry and convergent evolution. Immunogenetics 54: 830–841. 12671733 10.1007/s00251-002-0530-0

[65] Sidney J, Peters B, Frahm N, Brander C, Sette A (2008) HLA class I supertypes: a revised and updated classification. BMC Immunol 9: 1. 18211710 10.1186/1471-2172-9-1 PMC2245908

[66] Shiina T, Dijkstra JM, Shimizu S, Watanabe A, Yanagiya K, et al (2005) Interchromosomal duplication of major histocompatibility complex class I regions in rainbow trout (*Oncorhynchus mykiss*), a species with a presumably recent tetraploid ancestry. Immunogenetics 56: 878–893. 15696305 10.1007/s00251-004-0755-1

[67] Watterson GA (1975) Number of Segregating Sites in Genetic Models Without Recombination. Theoretical Population Biology 7: 256–276. 1145509 10.1016/0040-5809(75)90020-9

[68] Roach JC, Glusman G, Smit AFA, Huff CD, Hubley R, et al (2010) Analysis of Genetic Inheritance in a Family Quartet by Whole-Genome Sequencing. Science 328: 636–639. 20220176 10.1126/science.1186802 PMC3037280

