# Supplementary figures and images for: Selection and Phylogenetics of Salmonid MHC Class I: Wild Brown Trout (Salmo trutta) Differ from a Non-Native Introduced Strain
Source: PLoS One. 2013 May 7;8(5):e63035. doi: 10.1371/journal.pone.0063035 (PMC3646885; doi:10.1371/journal.pone.0063035)

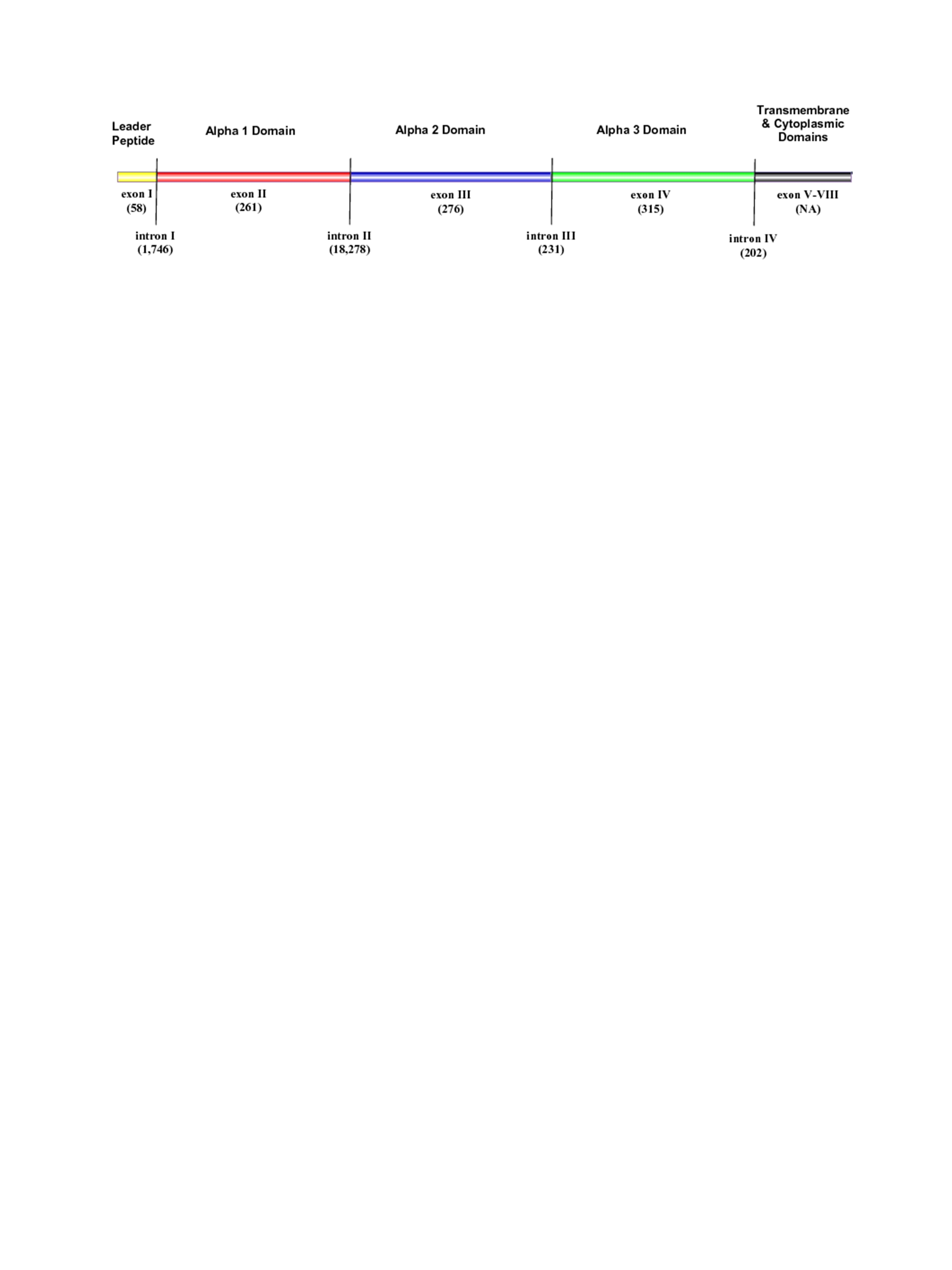

Supplement: Figure S1 — Salmonid UBA structure. Relevant structure of the salmonid UBA gene (after [66]) and based on the rainbow trout allele AF296362_Onmy-UBA*0501. Intron-exon organisation is shown with sizes for the relevant exons and introns in nucleotide base pairs given in parentheses. Note the large size of intron II between exons coding for the α1 and α2 domains. (TIF) [file pone.0063035.s001.tif]

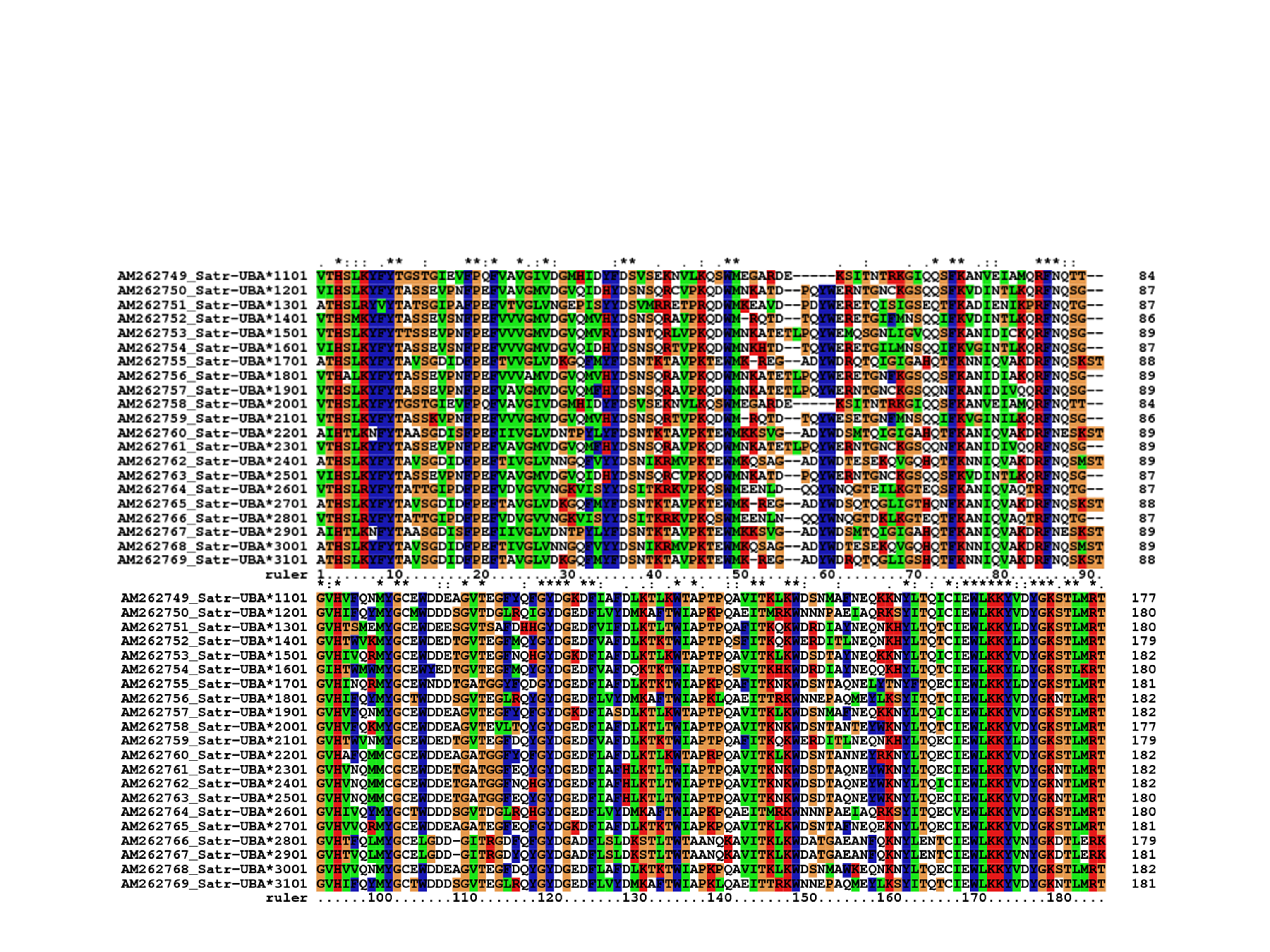

Supplement: Figure S2 — Novel Satr-UBA alleles. Amino acid alignment of novel Satr-UBA alleles described in this work. Accession numbers are included in each allele name. Sequences from the α1 domain (top) and α2 domain (bottom) are displayed together with the respective lengths of each sequence. (TIF) [file pone.0063035.s002.tif]

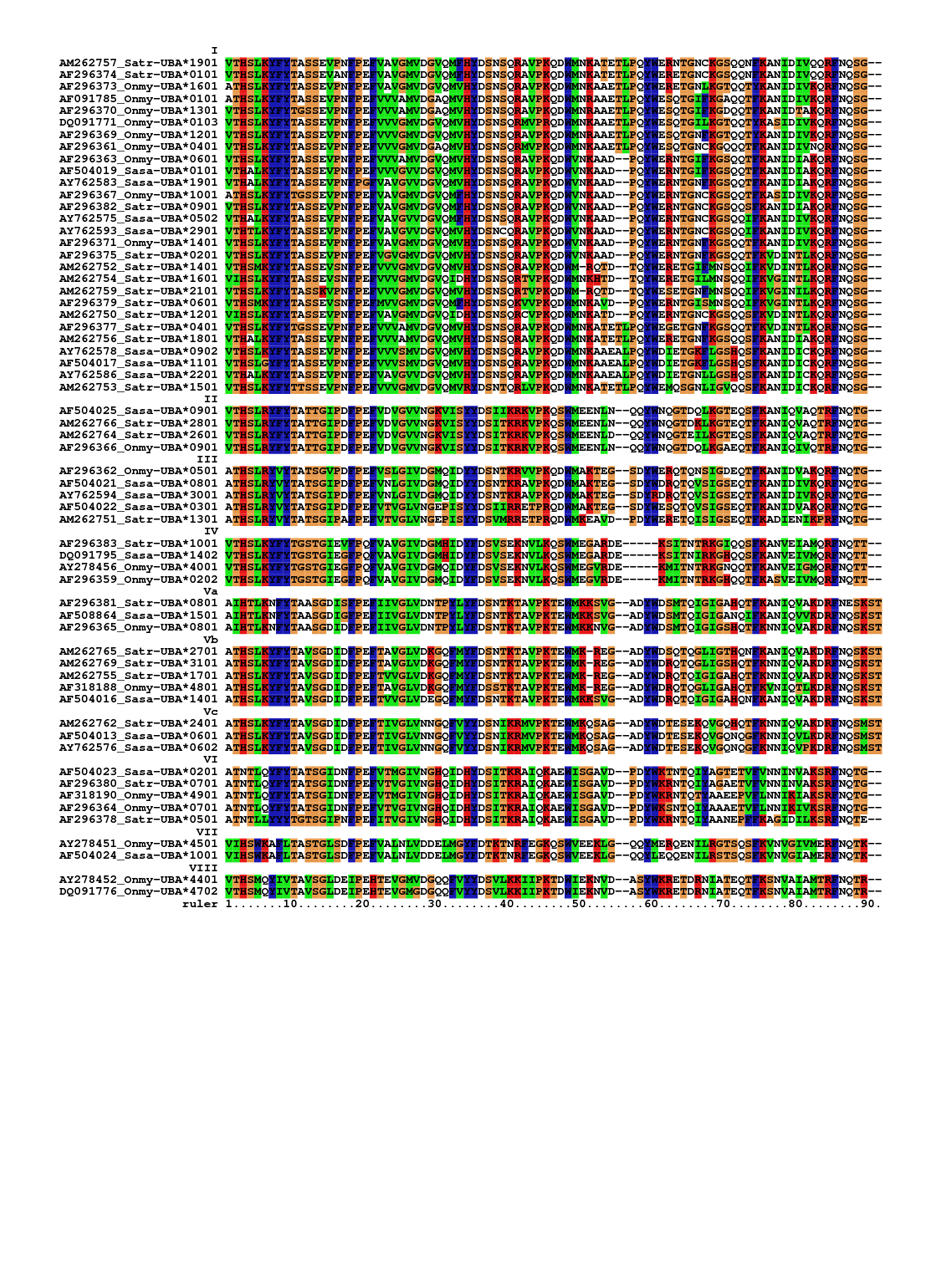

Supplement: Figure S3 — α1 sequence alignments. A) Representative salmonid UBA α1 domain amino acid sequence alignments capturing the diversity of variation within α1 lineages (roman numerals) and between lineages. We include Satr-UBA*1301 with α1 LIII sequences. When sites which were found to be under selection in OMEGAMAP were considered, it is noted that these fall into two categories, sites which are highly variable between lineages and sites which are highly variable both between and within particular lineages. (TIF) [file pone.0063035.s003.tif]

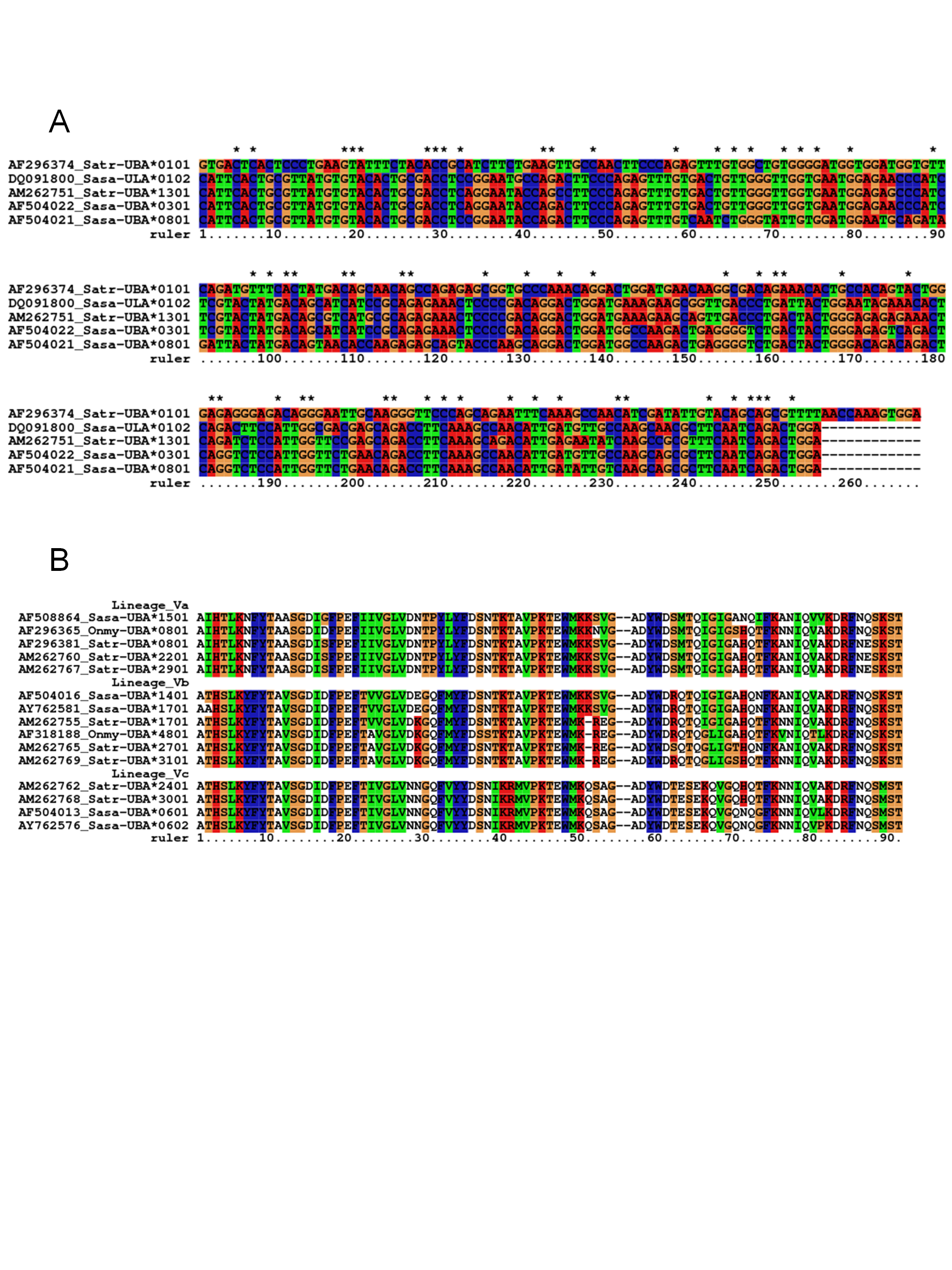

Supplement: Figure S4 — Alignments highlighting recombination in α1 lineages. A) Nucleotide sequences for Satr-UBA*1301, Sasa-UBA*0301, Sasa-UBA*0801, Sasa-ULA*0102 and an α1 LI sequence, the reference sequence, Satr-UBA*0101. Note that Satr-UBA*1301, Sasa-UBA*0301 and Sasa-ULA*0102 have very similar nt sequences between positions 1 and ∼136 whereupon Sasa-UBA*0301 is observed to abruptly demonstrate greater similarity to a typical α1 LIII sequence, Sasa-UBA*0301. Satr-UBA*1301 sequence similarity to the ULA sequence persists slightly longer but thereafter large numbers of nt differences are observed. This pattern is typical of recombination or gene conversion events occurring within the α1 domain. B) Amino acid alignments of α1 Lv lineages. Note the high degree of similarity between sequences from different species indicating that trans-species polymorphism is extensive in α1 Lv. Note that sequences in lineage LVb are more similar to sequences of LVc between aa positions 1-28 but more similar to LVa sequences in the remainder of the sequence. This pattern might be explained by an ancient recombination event (or events) between LVa and LVc sequences giving rise the poorly supported LVb clade. Notably, when α1 LVb sequences are removed from SPLITSTREE networks (data not shown), LVa and LVc sequences appear as distinct α1 lineages although sharing a more recent common ancestor than any other pair of lineages in the network. This suggests both that intradomain recombination between lineages is possible but also that it is more feasible between more closely related lineages. (TIF) [file pone.0063035.s004.tif]

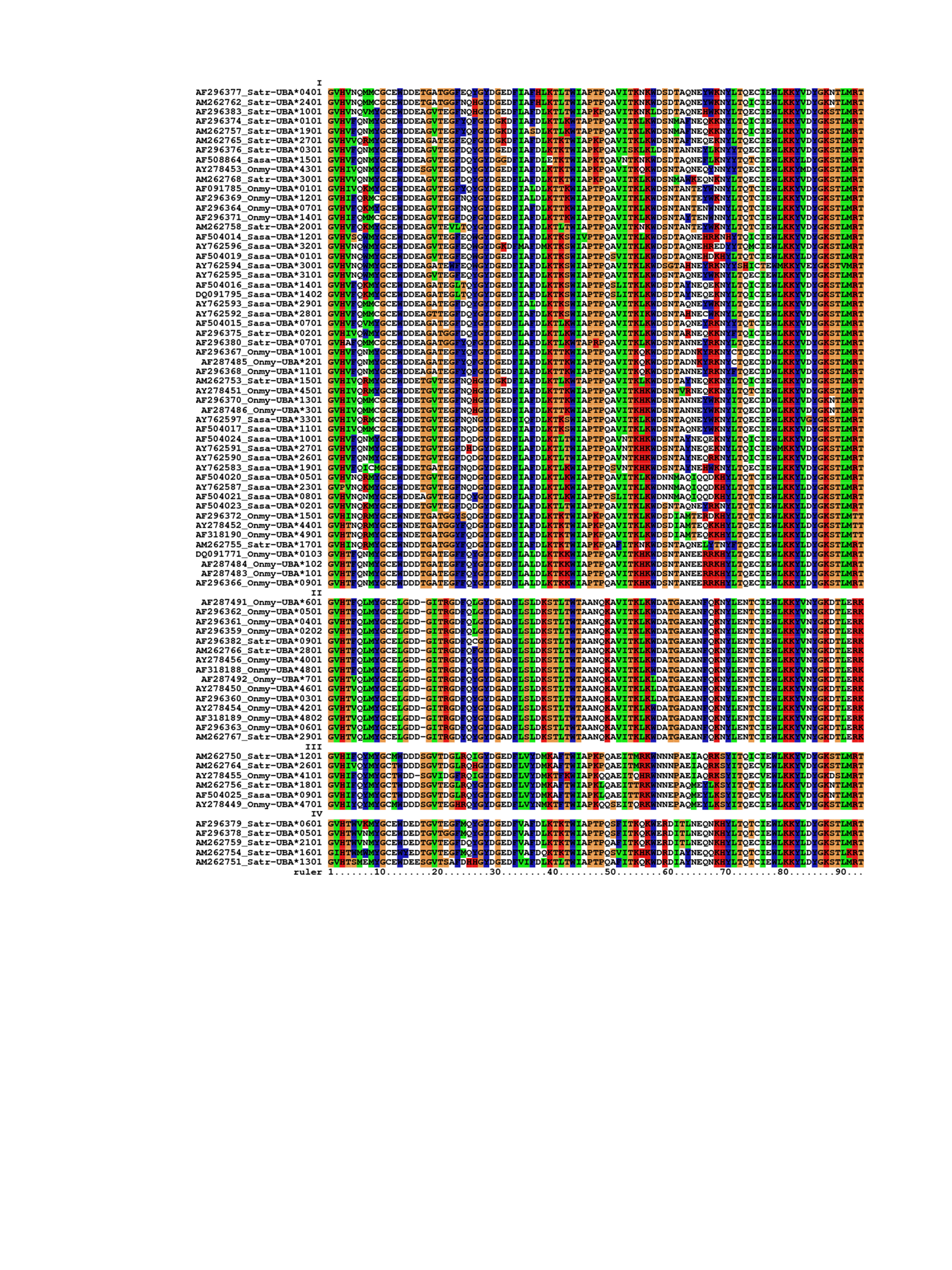

Supplement: Figure S5 — Alignment of representative salmonid UBA α2 domain amino acid sequence alignments showing the diversity of variation within α2 lineages (roman numerals) and between lineages. Sites found to be under selection in OMEGAMAP fall into two categories, sites which are highly variable between lineages and sites which are highly variable both between and within particular lineages. A notable feature of α2 diversity is the extensive and diffuse polymorphism within α2 LI. In contrast, a remarkable degree of conservation is observed within other α2 lineages. This may point to differences in selective pressures in different α2 lineages. (TIF) [file pone.0063035.s005.tif]

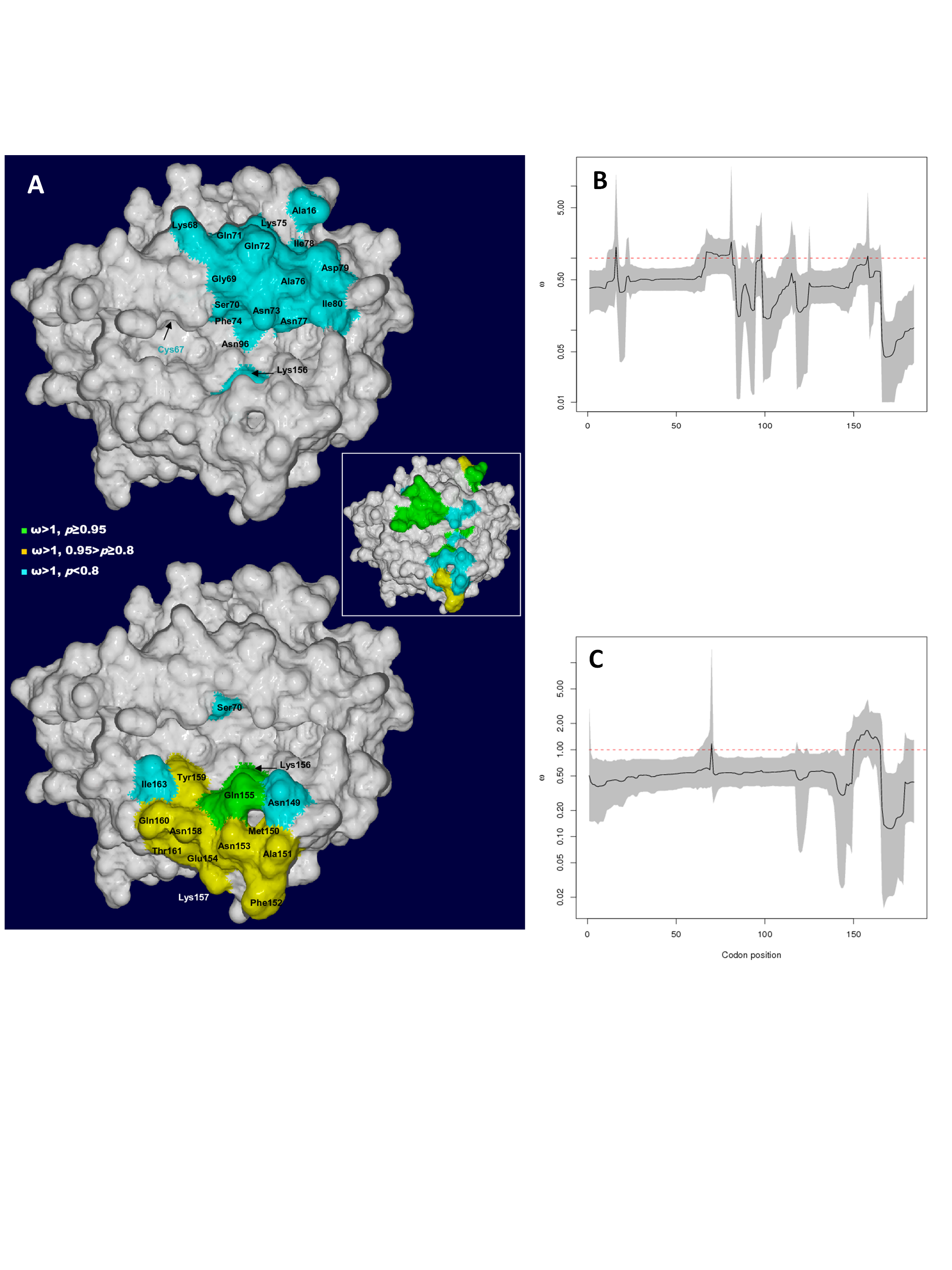

Supplement: Figure S6 — A) Model showing selected sites in the UBA protein for the Colorado River introduced populations of S. trutta population (top) and in the Colorado River O. mykiss population (bottom). For comparison, this information from the Srahrevagh River S. trutta population is also provided (inset, right, detail in Figure 1A). Clear differences in the distribution of selected sites in the peptide binding can be seen. B, C) Comparative plots of ω for the Colorado River S. trutta (B) and O. mykiss (C) populations. The pattern observed in the O. mykiss population is remarkably flat outside distinct diversifying selection foci at Ser70 and between Asn149 and Ile163. Highest Posterior Density (HPD) 95% confidence intervals are seen in grey about the plot line and are tight about means in all cases, suggesting confidence in the ω estimates. (TIF) [file pone.0063035.s006.tif]

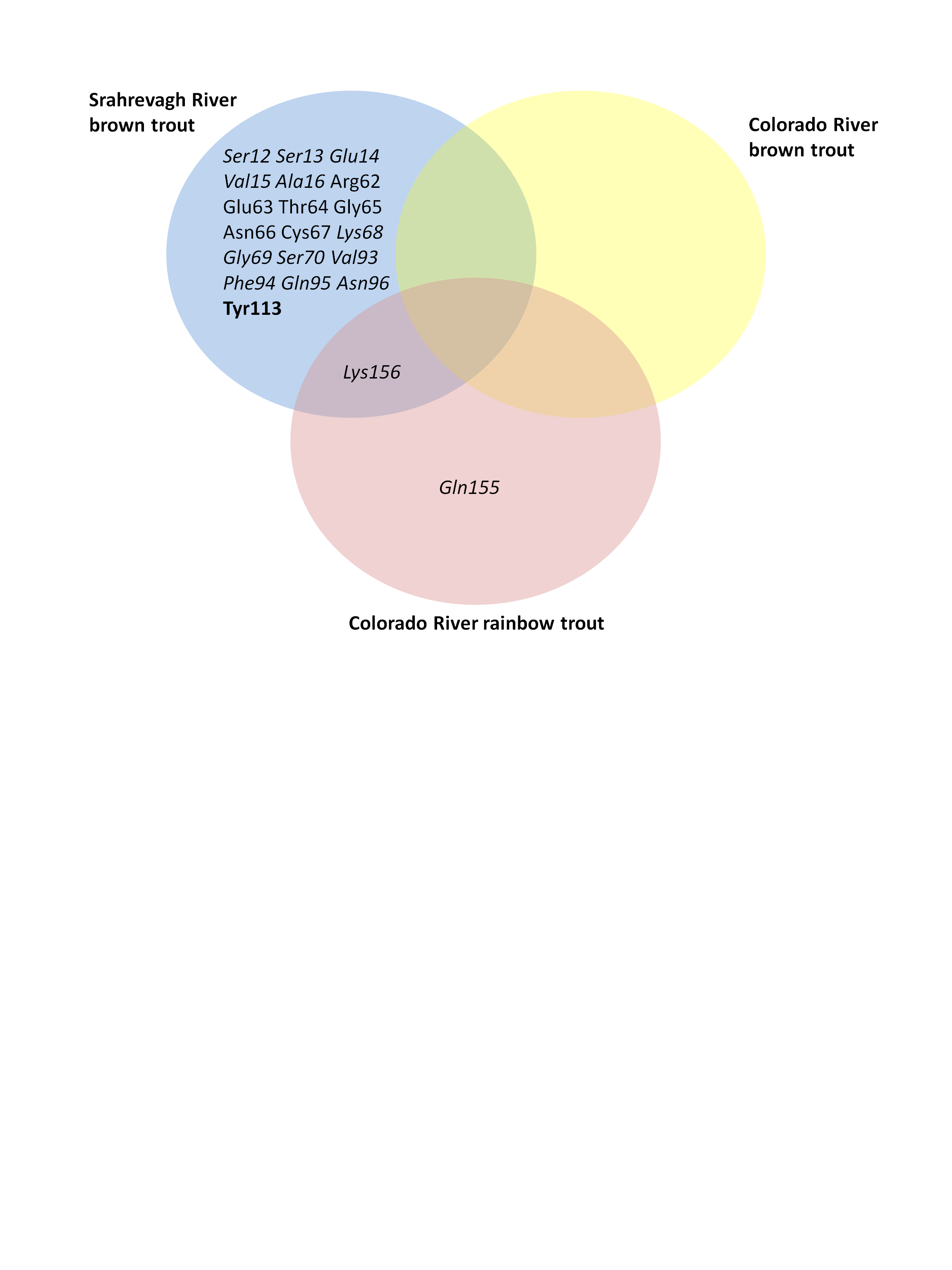

Supplement: Figure S7 — Selected sites in UBA. Venn diagrams of sites under selection identified in independent OMEGAMAP analyses of the three individual populations labelled. Significance levels of selection on residues: p<0.001 (bold), p<0.01 (normal) and p<0.05 (italics). (TIF) [file pone.0063035.s007.tif]

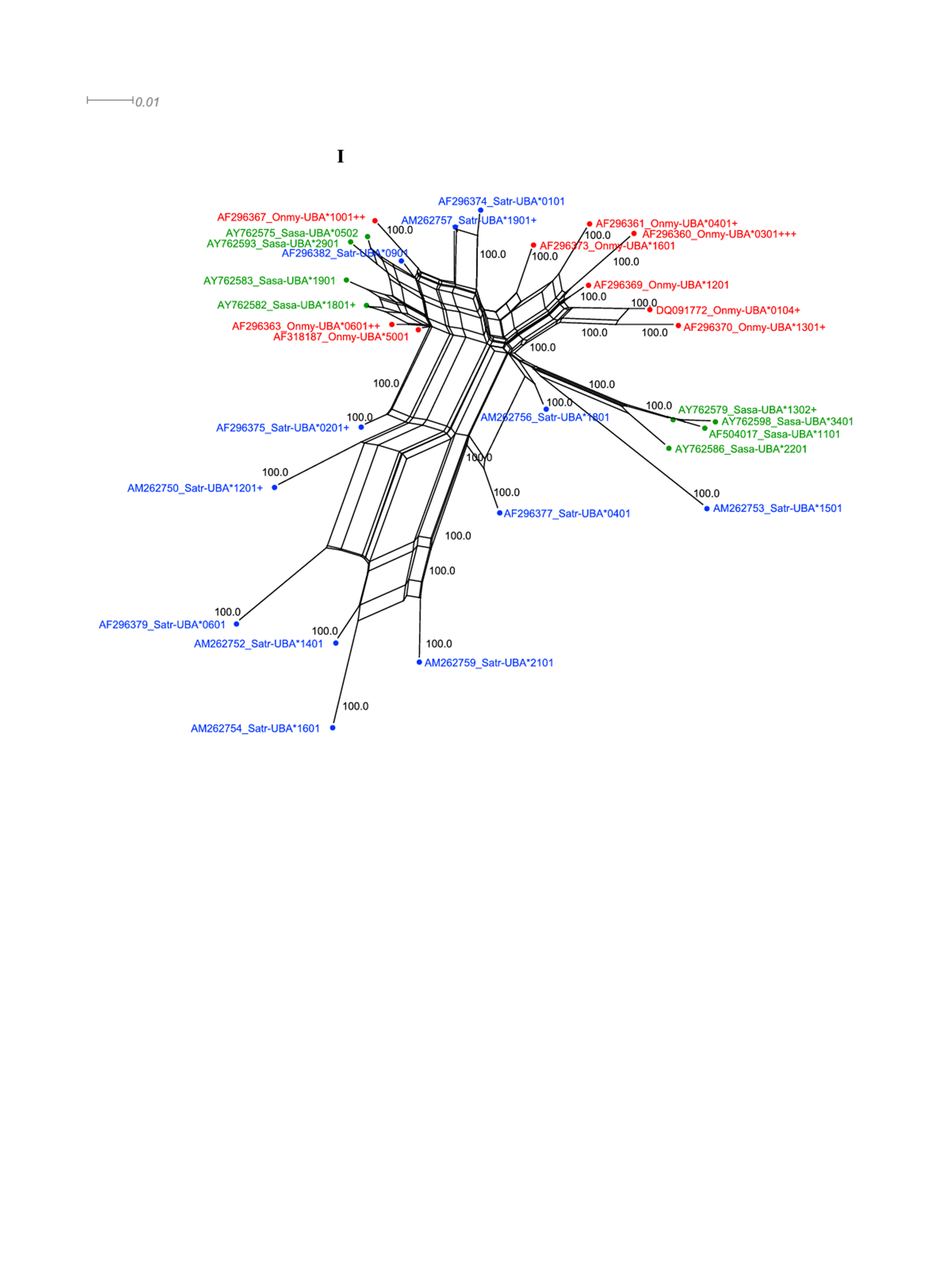

Supplement: Figure S8 — Phylogenetics of α1 Lineage I. α1 LI Large loops are observed in the network, particularly affecting Satr-UBA sequences, indicating recombination events. Other parts of the network are more treelike, suggesting a stronger role for point mutation. Each salmonid species demonstrates some species-specific diversification but trans-species polymorphism is observed even within this most diverse of α1 lineages. (TIF) [file pone.0063035.s008.tif]

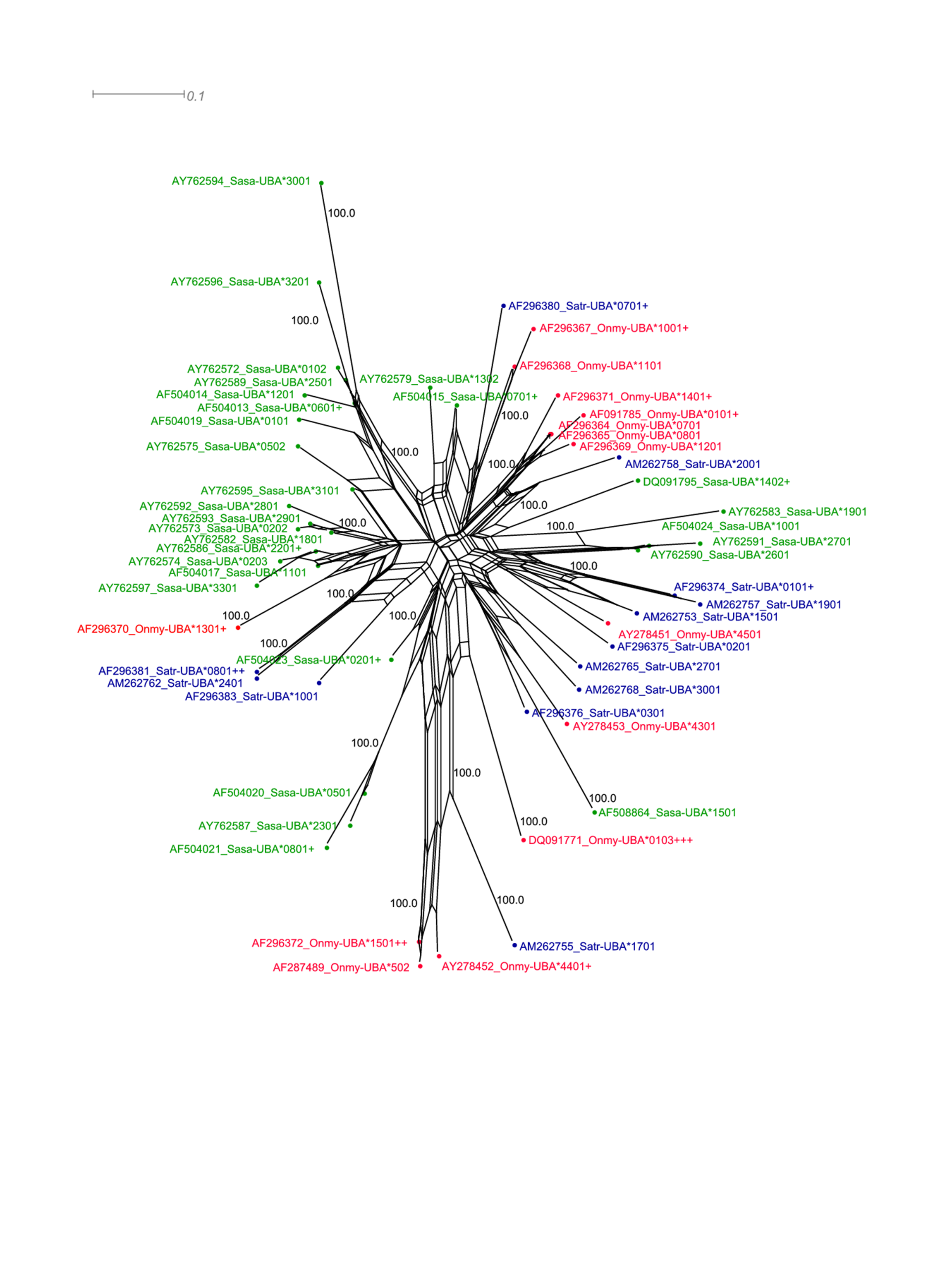

Supplement: Figure S9 — Phylogenetics of α1 Lineage I. The α2 LI network is typified by stellate radiation although incongruities may imply gene conversion, recombination or convergence also occurs. Trans-species polymorphism is observed although no sequences demonstrate a high degree of similarity. In other parts of the network, species-specific diversification is extensive, particularly for S. salar sequences. (TIF) [file pone.0063035.s009.tif]
