# Supplementary material for: Selection and Phylogenetics of Salmonid MHC Class I: Wild Brown Trout (Salmo trutta) Differ from a Non-Native Introduced Strain
Source: PLoS One. 2013 May 7;8(5):e63035. doi: 10.1371/journal.pone.0063035 (PMC3646885; doi:10.1371/journal.pone.0063035)
Supplement: Table S1 — Srahrevagh UBA allele frequency data. (DOCX) [file pone.0063035.s010.docx]

Table S1

| Allele | Frequency |
| --- | --- |
| *Satr-UBA**1101 | 3 |
| *Satr-UBA**1201 | 7 |
| *Satr-UBA**1401 | 2 |
| *Satr-UBA**1501 | 3 |
| *Satr-UBA**1601 | 3 |
| *Satr-UBA**1701 | 2 |
| *Satr-UBA**1801 | 2 |
| *Satr-UBA**1901 | 1 |
| *Satr-UBA**2001 | 1 |
| *Satr-UBA**2101 | 1 |
| *Satr-UBA**2201 | 1 |
| *Satr-UBA**2301 | 1 |
| *Satr-UBA**2501 | 1 |
| *Satr-UBA**2601 | 1 |
| *Satr-UBA**2701 | 1 |
| *Satr-UBA**3101 | 1 |
