# Supplementary material for: Selection and Phylogenetics of Salmonid MHC Class I: Wild Brown Trout (Salmo trutta) Differ from a Non-Native Introduced Strain
Source: PLoS One. 2013 May 7;8(5):e63035. doi: 10.1371/journal.pone.0063035 (PMC3646885; doi:10.1371/journal.pone.0063035)
Supplement: Table S2 — OMEGAMAP prior distribution parameter sets. Details of prior distribution sets. Prior A was used for analyses. (DOCX) [file pone.0063035.s011.docx]

| **Table S2 - Details of prior distribution sets. Prior A was used for analyses.** | | |
| --- | --- | --- |
| **Parameter** | **Prior A** | **Prior B** |
| Orderings | 10 | 10 |
| Burn-in | 25,000 | 25,000 |
| Iterations | 250,000 | 250,000 |
| Thinning interval | 100^th^ | 100^th^ |
| ω | 0.01 – 100 (inverse prior) | 0.01 – 100 (inverse prior) |
| ρ | 0.01 – 100 (inverse prior) | 0.01 – 100 (inverse prior) |
| μ | 0.3 (improper inverse) | 0.01-10 (inverse prior) |
| κ | 3 (improper inverse) | 0.01-20 (inverse prior) |
| Φ | 0.1 (improper inverse) | 0.05 (improper inverse) |
| Block model | 20 codons | 20 codons |
